# Emerging Concepts in Immune Thrombocytopenia

**DOI:** 10.3389/fimmu.2018.00880

**Published:** 2018-04-30

**Authors:** Maurice Swinkels, Maaike Rijkers, Jan Voorberg, Gestur Vidarsson, Frank W. G. Leebeek, A. J. Gerard Jansen

**Affiliations:** ^1^Department of Hematology, Erasmus University Medical Centre, Rotterdam, Netherlands; ^2^Department of Plasma Proteins, AMC-Sanquin Landsteiner Laboratory, Amsterdam, Netherlands; ^3^Department of Experimental Immunohematology, AMC-Sanquin Landsteiner Laboratory, Amsterdam, Netherlands

**Keywords:** immune thrombocytopenia, immune thrombocytopenic purpura, autoantibodies, CD8^+^ T cells, autoimmunity, ITP

## Abstract

Immune thrombocytopenia (ITP) is an autoimmune disease defined by low platelet counts which presents with an increased bleeding risk. Several genetic risk factors (e.g., polymorphisms in immunity-related genes) predispose to ITP. Autoantibodies and cytotoxic CD8^+^ T cells (Tc) mediate the anti-platelet response leading to thrombocytopenia. Both effector arms enhance platelet clearance through phagocytosis by splenic macrophages or dendritic cells and by induction of apoptosis. Meanwhile, platelet production is inhibited by CD8^+^ Tc targeting megakaryocytes in the bone marrow. CD4^+^ T helper cells are important for B cell differentiation into autoantibody secreting plasma cells. Regulatory Tc are essential to secure immune tolerance, and reduced levels have been implicated in the development of ITP. Both Fcγ-receptor-dependent and -independent pathways are involved in the etiology of ITP. In this review, we present a simplified model for the pathogenesis of ITP, in which exposure of platelet surface antigens and a loss of tolerance are required for development of chronic anti-platelet responses. We also suggest that infections may comprise an important trigger for the development of auto-immunity against platelets in ITP. Post-translational modification of autoantigens has been firmly implicated in the development of autoimmune disorders like rheumatoid arthritis and type 1 diabetes. Based on these findings, we propose that post-translational modifications of platelet antigens may also contribute to the pathogenesis of ITP.

## Introduction

Immune thrombocytopenia (ITP) is an autoimmune disease characterized by low platelet counts and increased bleeding risk (1–4). The initial event(s) leading to anti-platelet autoimmunity remains unclear, but strong evidence exists that autoantibodies and autoreactive CD8^+^ cytotoxic T cells (Tc) trigger enhanced platelet destruction and impair platelet production by megakaryocytes (MKs) in the bone marrow. We will briefly discuss the clinical aspects of this heterogeneous disease, followed by an overview of the mechanisms and pathways by which autoreactive B and Tc engage in anti-platelet immunity, with a particular focus on their specificity for platelet autoantigens. We will postulate a general model for ITP pathophysiology and finally highlight opportunities in ITP research, which can be derived from studies on other autoimmune diseases.

### Epidemiology and Clinical Features

Immune thrombocytopenia is a diagnosis of exclusion: patients who develop thrombocytopenia (defined as platelet counts below 100,000 platelets per microliter) with no clear underlying cause are currently diagnosed with (isolated) primary ITP (1, 4). Secondary ITP is defined as an ITP induced by other diseases or treatments. These include autoimmune disorders, lymphoproliferative disorders, infectious agents, transfusion, or induction by drugs, accounting in total for 20% of ITP cases (5, 6). In total, the incidence of ITP is approximately 1.9–6.4 per 100,000 children/year and 3.3–3.9 per 100,000 adults/year (6–8), and this number is increasing (6, 9). ITP can be classified in a transient form termed newly diagnosed ITP (up until 3 months), or persistent ITP (up until 12 months) that is more prevalent in children, or a chronic form (longer than 12 months) that does not resolve on itself and is more prevalent in adult patients (1, 6, 7). The acronym “ITP” should not be confused with the outdated definition of “idiopathic thrombocytopenic purpura” that has been used previously (1, 4). ITP is no longer considered an idiopathic disease and a proportion of patients do not present with purpura (see below). In this review, we discuss both adult and pediatric ITP studies and highlight discrepancies between both groups where necessary.

Bleeding symptoms in ITP patients typically present as either a mild form, such as bleeding in skin and mucosal regions, or a more severe, life-threatening form, such as bleeding in gastrointestinal or intracranial areas (6, 10). Patients with ITP have varying platelet counts as a result of the disease. Those with platelet counts above 50,000 per microliter rarely bleed, but below this “threshold” value, there are large differences in clinical phenotypes between patients that are as of yet unexplained (2, 3, 10). Platelet function testing appears successful in predicting bleeding risk in patients (11–13). However, no clear-cut diagnostic tools exist as associations between biomarkers and ITP remain limited, and no markers exist that may predict treatment responses (2, 3). The most common therapeutic options are based on immunosuppression [by corticosteroids, intravenous immunoglobulin (IVIg), or rituximab], or stimulation of platelet production [by thrombopoietin receptor agonists (TPO-RAs), see below].

### Platelet Life Cycle

On average, the human body produces around 100 billion platelets per day resulting in a concentration of ~150,000–400,000 platelets per microliter blood (14). Platelets circulate for approximately 7–10 days, slowly undergoing age-related changes in morphology, activation, and surface receptor density (15–18). Platelets are produced by MKs in the bone marrow (19). MKs are polynuclear cells that protrude extensions in the blood, termed proplatelets, and eventually bud off platelets from these extensions (20, 21). Recent findings show that MKs may also reside in the lung, facilitating platelet production in lung tissues (22), although the relevance of platelet production at this site is currently unclear.

Thrombopoietin (TPO) is the key hormone responsible for platelet production. It is primarily synthesized in the liver and promotes MKs to produce platelets in the bone marrow *via* the TPO receptor, Mpl (23–25). As newly made TPO is released in the bloodstream by hepatocytes, it is also incorporated into circulating platelets *via* Mpl. This constitutes an inhibitory feedback loop in which platelet counts inversely correlate with the amount of TPO reaching the bone marrow to stimulate new platelet production (23, 26). Recent evidence suggests that the Ashwell-Morrell receptor (AMR) on hepatocytes plays an important role in this physiological process. Normally, as platelets age terminal sialic acid is gradually lost from the surface, which exposes the underlying galactose residues. This allows for their clearance by the AMR (27). AMR-mediated platelet clearance triggers hepatic TPO transcription and translation, and new TPO is released (27). Several other physiological clearance mechanisms exist that control platelet numbers, such as platelet apoptosis (28) and possibly phagocytosis by αMβ2 integrins on hepatic and splenic macrophages [for a review, see Ref. (29)].

**Figure 1:**
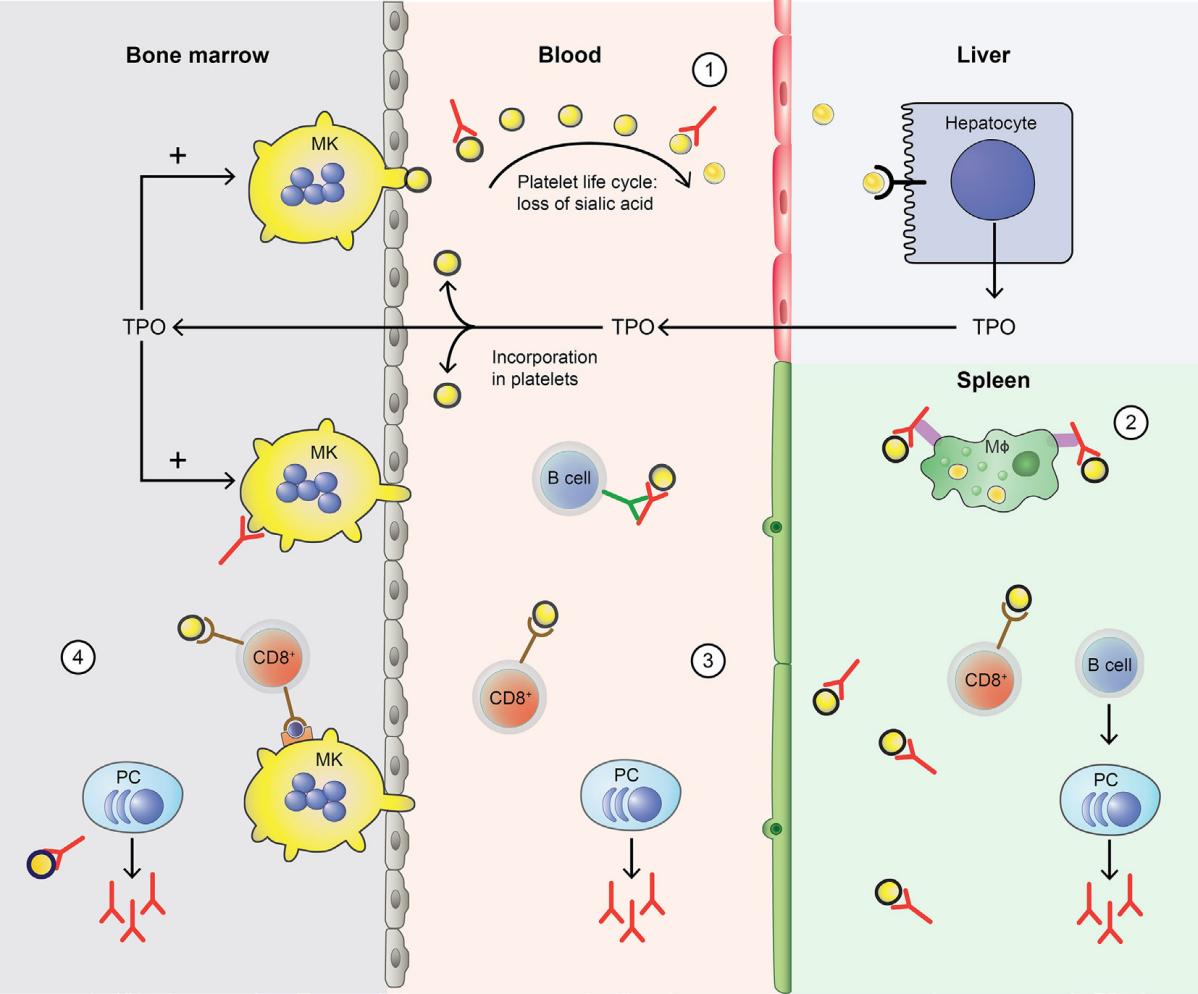
Disturbance of the platelet life cycle in immune thrombocytopenia (ITP). (1) Platelets (yellow) are normally produced by megakaryocytes (MKs, yellow) in the bone marrow. Aging platelets undergo apoptosis but also gradually lose terminal sialic acid from the surface (indicated by black circles). This allows for their clearance in the liver. Liver-mediated platelet clearance triggers hepatic TPO transcription and translation, and new TPO is released. This process is disrupted by autoantibodies in ITP, which are hypothesized to enhance platelet desialylation leading to enhanced clearance. (2) Macrophages (MF, green) can phagocytose platelets; meanwhile, platelet antigens are presented in the spleen to immune cells, such as CD4^+^ T helper (Th) cells. With CD4^+^ T cell help, B cells (B cell, dark blue) are able to differentiate into platelet-reactive plasma cells (PC, light blue) that can secrete autoantibodies (red). Cytotoxic T cells (Tc) (CD8^+^, red) can directly lyse platelets. (3) In peripheral blood, plasma cells and cytotoxic Tc further induce autoimmune responses against platelets. Cytotoxic Tc may also induce desialylation leading to enhanced clearance. In addition, platelet-reactive memory B cells may be present in the blood. (4) Plasma cells and cytotoxic Tc are also present in the bone marrow, where they can inhibit platelet production by targeting MKs.

In ITP, this normal platelet life cycle is disturbed by autoantibodies and platelet-reactive CD8^+^ Tc as summarized in Figure 1. Autoantibodies and CD8^+^ Tc may interfere with multiple aspects of the platelet life cycle, including their production and clearance that result in thrombocytopenia. In such thrombocytopenic conditions, the small amount of circulating Mpl-containing platelets often leads to high TPO levels (30, 31). Interestingly, only slightly elevated TPO levels are observed in ITP; likely because platelets with incorporated TPO are rapidly cleared (31). Therefore, one of the therapeutic options for ITP patients involves stimulation of the TPO receptor on MKs by TPO-RAs, which proves to be successful in many patients (32). Not all patients are equally responsive to TPO-RAs and poor responders likely suffer from a prolonged autoimmune response against platelets that cannot be resolved by increasing the platelet production.

### Genetic Risk Factors

As mentioned, autoreactive B and Tc have been firmly implicated in the pathophysiology of ITP. Consequently, many studies have reported associations between ITP and single nucleotide polymorphisms (SNP) in immunity-related genes. Polymorphisms in genes encoding specific cyto- or chemokines, such as interleukin (IL)-1, IL-2, IL-4, IL-6, IL-10, IL-17, TNF-α, TGF-β, and IFN-γ, have been associated with ITP (33–37). Several studies have also investigated whether specific HLA class I or II alleles are elevated in patients with ITP (38–45); current findings suggest that polymorphic sites within the HLA locus are not linked to ITP as studies have reported both significant and nonsignificant findings (37–44). The variation in studies could potentially be explained by small sample size, ethnic variability, or differences in diagnosis, yet does not allow to reach a consensus. New biomarkers such as miRNAs regulating levels of cytokines or other immune components are also increasingly recognized as potential risk factors for ITP (46). Classically, polymorphisms in Fcγ receptors (FcγRs) have been associated with the onset and pathogenesis of ITP (47–54) and are therefore further discussed below. Most of the reported association studies performed in ITP patients were conducted in small cohorts and in specific ethnic subgroups, and thus should be interpreted with caution. Additionally, many of the identified associations are not found in all patients and are commonly observed in other autoimmune diseases as well and are therefore general predisposing factors and not specific for ITP. Advances in (epi)genomics are likely to identify additional genetic risk factors for the development of ITP (55, 56).

### Environmental Risk Factors

For a long time, the occurrence of specific infections has been associated with ITP, particularly in children (5–7). Some of the most occurring and most studied infectious agents are *Helicobacter pylori* (57, 58), Hepatitis C virus (59, 60) and human immunodeficiency virus (61–67). Evidence also exists for Cytomegalovirus (68, 69), Epstein Barr Virus (69), and some other viruses (70, 71). Although individual cases of ITP have been reported after vaccination, this is exceedingly rare (72, 73). One of the suggested mechanisms by which infections lead to autoimmunity is the occurrence of molecular mimicry. In this case, viral proteins resemble platelet receptors to evade the immune system (74). In case of an immune response against these viral proteins, cross reactivity may occur against platelet receptors, which subsequently lead to autoantibodies specific for both the viral protein and platelet receptors. This could explain the initiation of ITP in some cases (60–63, 66), which can be resolved by clearance of the infectious agent after which autoantibodies diminish (57, 58). Besides a transient decrease in platelet counts, infections sometimes elicit strong immune responses that can perpetuate and develop into chronic ITP, resulting in sustained platelet clearance.

Toll-like receptors are present on various innate immune cells, including platelets, and are suggested to mediate some of the microbial-platelet interactions that can trigger and/or aggravate autoimmunity (75). Immune-mediated thrombocytopenia may also occur as a result of other autoimmune diseases, drugs, transfusion, and in lymphoproliferative disorders (76, 77). Often, these cases are also diagnosed as secondary ITP, but may greatly differ in etiology. As our review focuses on primary ITP, we refer readers to Ref. (77) for more information on the underlying pathophysiology of these forms of secondary ITP.

## Etiology

### Autoantibodies

**Figure 2:**
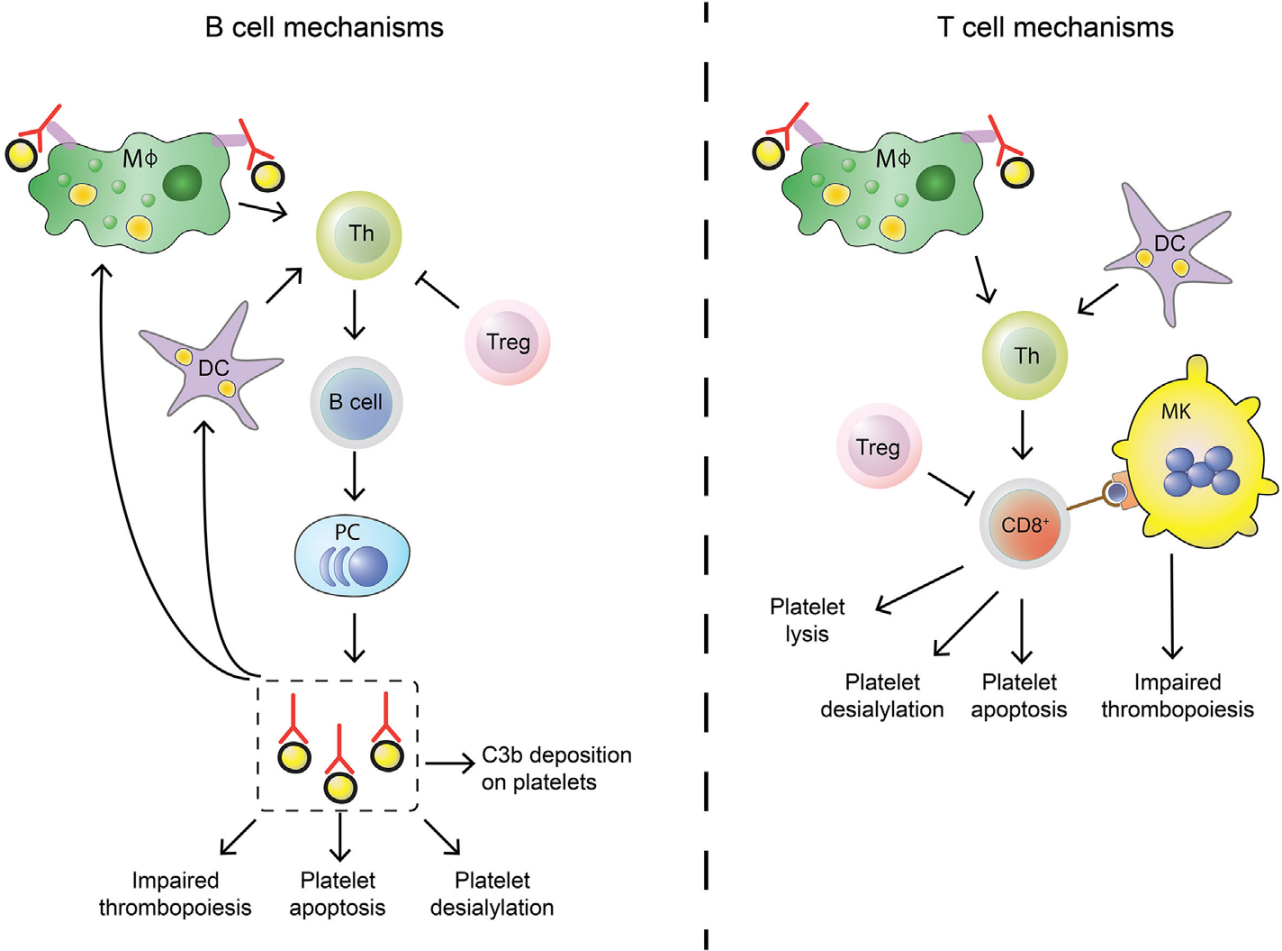
Differences in B cell and T cell mechanisms in immune thrombocytopenia (ITP). B cells (left) differ from cytotoxic T cells (Tc) (right) in their autoimmune response against platelets in ITP. Stimulation of the adaptive immune response is similar: splenic macrophages (green) and dendritic cells (DCs, purple) can phagocytose platelet fragments to present to T helper (Th, light green) cells. Th cells are able to induce B cell development into autoantibody secreting plasma cells and can also stimulate cytotoxic Tc effector mechanisms. This process is regulated by regulatory Tc (Treg, pink), but regulatory T cell levels are imbalanced in ITP patients which leads to insufficient control of the autoimmune response. Shared effector functions of B cell-produced autoantibodies and cytotoxic Tc include impairing thrombopoiesis by targeting megakaryocytes (MKs), inducing platelet apoptosis and enhancing platelet desialylation. Autoantibodies can further stimulate C3b deposition on platelets to initiate complement activation, while cytotoxic Tc can directly lyse platelets.

In approximately 60% of all ITP patients, autoantibodies are found, predominantly against platelet glycoprotein (GP) IIb/IIIa (~70%) and/or the GP Ib–IX–V complex (~25%) (78–81). Antibodies against GPIa–IIa or GPVI are also detected in sporadic cases (~5%) (80, 82, 83). While it is not entirely clear how autoantibodies against platelet antigens are generated, their effect on platelet clearance and production have now been fully elucidated (Figure 1). When microbial-antigens mimicking platelet autoantigens, or the platelet antigens themselves, are presented to B cells, these can develop into autoantibody-secreting plasma cells. The spleen has been implied as an organ where immune cells are primarily presented with platelet autoantigens, and where platelet clearance takes place most (84, 85). Particularly splenic macrophages and dendritic cells (DCs) can present platelet antigens to T helper (Th) cells that provide help to B cells that differentiate into antibody-secreting plasma cells (86, 87). Plasma cells secreting platelet-reactive autoantibodies are present in peripheral blood and bone marrow, where they can further generate autoantibodies that can sequester platelets and MKs (88–90). In addition, memory B cells activated in the spleen are also released in the circulation (Figure 2) (85). Autoantibodies accelerate platelet clearance by removal *via* splenic macrophages and DCs (87), complement deposition (91–93) and platelet apoptosis (94), or by inhibiting megakaryocytic platelet production (88–90).

### Autoantibody and B Cell Classification

Initial studies investigating autoantibodies in ITP identified high levels of platelet-associated IgGs (PAIgGs) in nearly all patients, and they were soon thought to be the causative factor of the autoimmune response. However, it was found that PAIgGs bound nonspecifically to platelets and were detected in other non-ITPs as well (95), likely because platelets themselves can bind circulating IgG *via* FcγRIIa (96). PAIgGs thus proved to be a poor predictor of the disease [for a review, see Ref. (95)]. Although it is interesting that PAIgGs levels are higher in ITP and other thrombocytopenic patients (consisting of different IgG subclasses compared to healthy individuals), their usefulness in investigating ITP remains limited and can be largely subscribed to the state of thrombocytopenia rather than the autoimmune conditions. Following the introduction of the MAIPA and immunobead assays in 1987 (79, 97), investigators were able to detect and further study platelet-specific autoantibodies in ITP (98, 99).

Most autoantibodies found in chronic ITP patients are of the IgG class, but IgM and sporadically IgA antibodies are also detected (100–102). IgM antibodies were shown to fix complement on platelets which could facilitate clearance, but this has not been further investigated; IgG autoantibodies seem to be the main mediator of antibody-driven autoimmunity (100). Most prevalent are IgGs of the IgG1 subclass, and while IgG2, IgG3, and IgG4 subclass autoantibodies can be also found in patients, they are often accompanied by IgG1 antibodies (103, 104). Autoantibody allotypes and Fc-glycosylation are important determinants in antibody-mediated immunity and immunological disorders related to ITP (105–107), yet have been scarcely investigated.

In the majority of ITP patients, B cells producing platelet-binding antibodies have been identified in clinical samples from different sources, such as peripheral blood, spleen, and bone marrow (108–115). However, not all patients have platelet-reactive B cells (108, 109, 112–114), suggesting that B cell independent autoimmune mechanisms (such as CD8^+^ T cell mediated autoimmunity) exist. A landmark study by Roark and co-workers employed repertoire cloning to clone platelet autoantibodies from the spleen of two patients with chronic ITP (110). Sequence analysis of Ig heavy chain arrangements revealed that these anti-platelet antibodies evolved from a restricted number of B cell clones and provided evidence for extensive modification of heavy chain segments by somatic hypermutation (110). Overall, these findings provide evidence for a CD4^+^ T cell-driven antigen-specific response in patients with ITP. Evidence for the selective incorporation of the VH3-30 variable heavy chain gene segment was noted in this study providing additional evidence for a restricted, oligoclonal B cell response targeting a limited number of epitopes on platelet antigens in ITP patients (110).

### Autoepitope Specificity of Antibodies

As the predominant source of epitopes for autoantibodies in ITP, the GP IIb/IIIa receptor, or integrin α_IIb_β_3_, has been studied most frequently. Reports have shown that autoantibodies can bind epitopes in both the extracellular- and cytoplasmic domain of GPIIb/IIIa (80, 116). However, autoantibodies targeting the cytoplasmic domain are likely to be generated during platelet destruction rather than being pathogenic, but their significance remains unclear (80). Subsequent studies have shown that autoantibodies particularly bind to the IIb subunit (117, 118), or contradictory, the IIIa subunit of the dimer (119). Eventually, several investigators have demonstrated that specific portions of the protein are preferred autoepitopes in ITP, often near ligand binding sites (81, 120). The vitronectin (α_v_β_3)_ receptor shares the β_3_ integrin with GPIIb/IIIa and was shown to be an important autoantigen in ITP as well (121). However, this has not been further investigated.

Less is known about relevant autoepitopes on GP complex Ib-IX-V, although most antibodies are directed against the GPIb part of the receptor complex (78, 98, 122). Interestingly, patients with autoantibodies against GPIb are often less responsive to immunosuppressive therapy with corticosteroids or IVIg when compared to patients with GPIIb/IIIa autoantibodies (122–124). This could be explained by specific epitopes on GPIb, relative receptor abundance on the platelet surface or differences between both protein complexes.

### B Cell Help by CD4^+^ T Cells

B cells require help by CD4^+^ Tc to efficiently develop into antibody-secreting plasma cells (Figure 2). As the development of autoantibodies is a hallmark of ITP, several studies have explored the involvement of CD4^+^ Tc in the pathogenesis of ITP. Initial observational studies showed that cytokines necessary for Th functions (such as IL-2, IL-10, IFN-γ) are increased in ITP patients (125, 126). Further evidence came from studies that identified a T cell imbalance in ITP: patients have a disturbed Th1/Th2 subset ratio, which trends toward a Th1 phenotype (127–129). Both rituximab and splenectomy seemed to resolve this polarization in responding patients, indicating the importance of balancing different populations of CD4^+^ Tc in ITP (128, 130). Pioneering work by Kuwana and co-workers have provided firm evidence for the presence of auto-reactive CD4^+^ Tc that target epitopes on GPIIb/IIIa (131, 132).

Recently, pro-inflammatory Th17 cells have emerged as a critical player in development of autoimmunity (133). Higher levels of Th17 cells were observed in several ITP cohorts (134, 135), but not in all studies (136). Several studies have found higher levels of both Th1 and Th17, compared to Th2 (137–141). The potential involvement of another subset of Th cells, Th22, was also investigated in ITP. Th22 cells typically promote protective and regenerative responses with predominant effects on epithelial cells (142–144). Increased levels IL-22 and elevated levels of the Th22 T cell subset have been observed in patients with ITP suggesting a role for this population of Tc in ITP pathogenesis (145, 146). In line with its established role in B cell help, splenic follicular Th (T_FH_) cells have also been implicated in the pathogenesis of ITP (147). These findings show that multiple Th populations including Th1/Th17/Th22/T_FH_ contribute to the pathogenesis of ITP (127, 129, 135, 137, 145–147). We anticipate that the observed skewing toward Th1/Th17/Th22/T_FH_ populations is not specific for ITP as similar Th polarization profiles are observed in other autoimmune diseases.

### CD8^+^ T Cells

Besides autoreactive B cells, CD8^+^ Tc have also been implicated in ITP pathogenesis (126, 148, 149). Evidence from association studies shows that patients with ITP more often present with polymorphisms in CD8^+^ related cytokines (126, 150, 151), have increased granzyme levels (152), and have imbalanced ratios of CD8^+^ Tc cell subsets (137, 140). As CD8^+^ Tc are also dependent on help of CD4^+^ Th cells to efficiently perform effector functions, the polarization of CD4^+^ Th cells probably also affects the CD8^+^ Tc cell response (137, 140).

T cells are part of cell-mediated immunity and have different effector functions compared to antibody-secreting B cells. In ITP, B cells and Tc thus elicit different forms of anti-platelet immunity (Figure 2). CD8^+^ Tc have been shown to directly lyse platelets (148, 153–155), induce platelet apoptosis (153), and inhibit thrombopoiesis by MKs (156). CD8^+^ Tc can further inhibit platelet production by inhibiting MK apoptosis (157).

Increased levels of CD8^+^ Tc were found in patients without autoantibodies (154), suggesting that CD8^+^ Tc cell-mediated autoimmunity can be elicited separately from autoantibody-mediated autoimmunity. Evidence of a T cell response separate from antibody-mediated autoimmunity was further shown in ITP patients who did not respond to the anti-CD20^+^ B cell-depleting antibody rituximab, in whom increased levels of splenic CD8^+^ Tc were detected (158). In contrast, CD8^+^ Tc were found to be protective and required for effective steroid therapy in a murine model of ITP, although these findings are counterintuitive and not supported by observations in other autoimmune diseases (159).

It is unclear how the B cell depletion and repopulation effects of rituximab alter T cell subsets in responding patients. Possibly, the altered cytokine environment as a result of B cell depletion affects T cell subsets, as the B-T cell interplay is essential in a systemic autoimmune response (160). A recent study showed that rituximab could suppress murine CD8^+^ T-cell mediated immune responses (161), suggesting that B cells may regulate the CD8^+^ T-cell response in ITP. In fact, ITP patients present with lower levels of regulatory B cells (162). However, the effect of rituximab treatment in ITP remains difficult to interpret as B cell depletion may also affect CD20^+^ regulatory B cells, which can secrete IL-10 and other suppressive cytokines to induce immune tolerance (163), as suggested previously.

As of yet, the target peptides expressed on MHC class I recognized by platelet specific CD8^+^ Tc have not been identified. Interestingly, no clear HLA association is found in ITP patients (38–45), as opposed to other autoimmune diseases. In *H. pylori*-mediated ITP, HLA associations were also unclear (114, 164). Platelets are capable of presenting non-renewable MK-derived peptides on MHC class I, and it is likely that these peptides are being recognized by CD8^+^ Tc that develop in patients with ITP (165). More recently, it was proposed that platelets have the propensity to activate naïve CD8^+^ Tc and that platelets can present pathogen-derived peptides in the context of MHC class I (166). In this context, it is interesting to note that following dengue infection the MHC class I density on platelets increases, suggesting an active role of platelets in combatting infections (166, 167). Under resting conditions, platelets do not express MHC class II molecules on their surface, but several reports suggested platelets to express MHCII complexes during infection (164, 168). Whether antigen presentation on MHC class I by platelets has a role in the pathogenesis of ITP has not been demonstrated. In view of the established role of CD8^+^ Tc in this autoimmune disorder, this will be an interesting area for further research.

### Regulatory T Cells

Tregs are a crucial checkpoint to limit immunity and secure immune tolerance. As such, they are important regulators that keep both B- and T cell-mediated autoimmunity in check (Figure 2). The importance of Tregs for the pathogenesis of ITP is evidenced by their reduced numbers and function in patients (169–173). The pivotal role of immune regulation in ITP, particularly by Tregs, was further shown by phenotypic and Treg profiling studies of treated versus untreated ITP patients. Treatment with corticosteroids and/or rituximab in responding patients both improved Treg levels as well as their activity (130, 174–178), indicating that loss of tolerance is essential for the pathogenesis of ITP. In an experimental murine model of ITP, Tregs were retained in the thymus. This was resolved by IVIg treatment, which normalized Tregs in the periphery (179). Additionally, transferring retained thymocytes delayed the onset of ITP, suggesting Tregs actively prevent ITP development at least in mice (179). Interestingly, TPO-RAs improved Treg activity indicating that platelets could directly or indirectly play a regulatory role in ITP by affecting Treg levels (175). As such, it is clear that ITP patients present with lower Treg levels which are restored upon successful treatment (see above). However, it is still unclear whether restoring Treg functionality directly alleviates the disease or is simply a marker of restored immune tolerance. Potentially involved pathways are further discussed below.

Tregs can interact with DCs to induce a tolerogenic phenotype. Two studies found that the interplay between Tregs and DCs is impaired in ITP (178, 180). As Treg levels are lower in ITP, this leads to a reduced expression of immunomodulatory enzyme indoleamine 2,3-dioxygenase 1 (IDO1) by DCs, and increased levels of mature DCs that can present (auto)antigens to other immune cells (178, 180). The important role of tolerance induction by DCs in ITP was further suggested by another study, in which IVIg was shown to mediate its effect *via* DCs in a murine model (181). The interplay between Tregs and DCs and immunomodulation *via* IL-10 is not only important in ITP but was also found essential in antibody-mediated acute lung injury (182, 183). As such, the Treg-DC-axis may be particularly important in autoantibody-mediated ITP, but this remains to be investigated.

### Other Immune Cells

Several other immune cells may modulate autoimmune responses in ITP but have been investigated sparsely. Neutrophils have been found to line MKs in ITP bone marrow (184), but their role in ITP has not been further investigated. A subset of CD16^+^ monocytes derived from patients with ITP has shown to promote the proliferation of IFN-γ^+^ CD4^+^ Tc (185). Shifts in the balance of inhibitory and activating FcγRs were observed on monocytes following treatment with high-dose corticosteroid dexamethason as well as following *H. pylori* eradication in ITP (186, 187) (further discussed below). Additionally, they were found to be involved in T cell development (185). Both increased and decreased levels of NK cells have been found in ITP patients (188–190). The significance of these observations is unclear since NK cells are not able to lyse platelets (148).

Finally, platelets themselves may be able to affect the autoimmune response in ITP, as they are increasingly recognized as mediators of immunity and inflammation [for a recent review, see Ref. (191)]. Evidence for such an autoregulatory loop was found in ITP patients responding to TPO-RAs, who not only had increased platelet counts but also correlating higher TGF-β plasma levels (175). Presumably, increased plasma TGF-β levels derive from an increased platelet mass (175). Furthermore, TPO-RAs reduced both autoantibody and T cell responses in a mouse model, which also lead to elevated TGF-β plasma levels (192). Interestingly, TPO-RAs may induce remission in a subset of patients whom then no longer needed therapy to maintain platelet levels (193–195). This would imply that immune tolerance can be restored in certain patient subsets by enhancing platelet numbers. Another mechanism by which platelets regulate immune responses occurs *via* CD40L. Activated Tc can stimulate B cell proliferation and differentiation *via* CD40L interactions with CD40 on B cells (196). Platelets normally express CD40L only upon activation, but higher baseline levels are observed in ITP patients (13). Furthermore, activated platelets from ITP patients were shown to stimulate autoreactive B cells by CD40L (197). Interestingly, CD40L inhibition was successful in suppressing T cell-assisted B cell-mediated autoantibody production in ITP, even in treatment of refractory ITP (198, 199). However, whether this is similarly successful affecting a potential B cell-platelet interaction remains unknown.

## Pathways Involved in Platelet Clearance

### FcγR-Mediated Eradication of Platelets

FcγRs have long been implicated in ITP etiology. These receptors are differentially expressed on immune cells and are the primary receptor for IgG. FcγRs mediate different functions, including phagocytosis, antibody dependent cellular cytotoxicity, and release of cytokines [reviewed in detail in Ref. (96)]. Most FcγRs are involved in activating the immune system, whereas FcγRIIb is the only inhibitory FcγR. Platelets only express FcγRIIa on their surface, while myeloid cells, such as granulocytes, monocytes, macrophages, and DCs express several FcγRs (96). In liver and particularly spleen, monocytes and macrophages have been suggested to bind and phagocytose Ig-opsonized platelets by FcγRs, explicitly contributing to platelet clearance and autoantigen presentation (85, 87). As such, polymorphisms in several FcγRs have been associated with ITP (47–54). The low affinity FcγRIIa, FcγRIIIb on granulocytes, and FcγRIIIa on NK cells, monocytes, and macrophages all contain SNP that affect binding affinity to IgG (200).

For FcγRIIa, one polymorphism at position 131 (R/H, with H having higher affinity) most strongly or exclusively affects IgG2 binding (200), and the higher-affinity allele was found to be associated with ITP (48, 51–54). However, these studies had inconsistent outcomes. A recent meta-analysis indicated that the R131H polymorphism might be associated with a subgroup of childhood-onset ITP, but this should be interpreted with caution (54). In accordance with the notion that FcγRIIIa^+^ splenic monocytes are particularly important for the clearance of platelets, only the higher affinity-allele of the FcγRIIIa polymorphism at position 158 [F/V, with 158 V having higher affinity for IgG1 and IgG3 (200)] has been found to be associated with ITP (48, 50–53). Intriguingly, one study found that a polymorphism in the transmembrane region of the inhibitory FcγRIIb (232I/T) is associated with the onset of newly diagnosed ITP in children (49). This polymorphism (232T) has been found to negatively affect the capacity of this receptor to downregulate immune responses (201) and could point at an immunomodulatory role of FcγRIIb. Intriguingly, eradication of *H. pylori* (a potential molecular mimicry causative of the onset of ITP) was found to shift monocyte FcγR expression toward an inhibitory FcγRIIb phenotype (187). Finally, the FcγRIIc has also been associated with ITP (50). FcγRIIc is a pseudogene in most individuals, but having FcγRIIc most likely predisposes individuals to stronger immune responses (202, 203). While the extracellular IgG-binding domain of FcγRIIc is identical to the inhibitory FcγRIIb, the intracelllular tail is identical to FcγRIIa and contains an activating motif (202). Due to the proposed expression of FcγRIIc on B cells, it may downregulate the negative feedback provided by FcγRIIb (202). Interestingly, FcγRs are known to crosstalk with Toll-like receptors, particularly during bacterial infections. This leads to T cell polarization (204), but it is unclear if this crosstalk is in any way relevant for platelets and/or in the context of ITP. Considering the strong correlation with infections in the onset of ITP, investigating the FcγR-TLR crosstalk could be interesting.

Additional evidence that FcγR-mediated pathways are important in ITP pathogenesis was shown by the therapeutic use of IVIg, which may bind FcγRs by its Fc-portion (205), and is one of the successful cornerstone treatments for ITP to rapidly increase platelet counts. It was recently shown that IVIg does not modulate FcγR expression directly but inhibits the phagocytic capabilities of splenic macrophages (206). In addition, a previous pilot study has also shown that Syk-inhibitors, which affect downstream FcγR signaling, can improve ITP (207). While IVIg does not work in all patients, the efficacy may be predicted by specific FcγR polymorphisms (208). As such, various FcγR polymorphisms provide the most compelling evidence that genetics may affect ITP, both by predicting higher risk of disease development and treatment outcomes. In addition, the role of FcγRs on platelets and other immune cells has now been firmly implicated in ITP pathogenesis. Nevertheless, FcγR-independent mechanisms may exist as well.

### FcγR-Independent Eradication of Platelets

A recent study has implicated a FcγR-independent pathway in an experimental mouse model (209), which was hypothesized to occur simultaneously aside FcγR-mediated clearance by splenic macrophages. Autoreactive antibodies against GPIb were hypothesized to induce platelet activation and degranulation, which leads to sialidase release (210). This induces desialylation of platelet membrane glycans, which can subsequently lead to recognition of platelets by the AMR in the liver thereby accelerating platelet clearance (27, 209). Interestingly, there are a few cases of ITP patients with abnormal platelet surface sialic acid levels (211, 212). Oseltamivir, which is a sialidase inhibitor used to treat influenza, has been found to increase platelet sialic acid content (213) and in two cases was successful in ameliorating thrombocytopenia whereas conventional therapy was not (212, 214). Platelet desialylation was also found to correlate with non-responsiveness to first-line therapies in ITP (215). Finally, CD8^+^ Tc have also been suggested to induce platelet desialylation and to facilitate platelet clearance similar to the earlier mentioned mechanisms (155). While the importance of sialic acid in the platelet life cycle has long been established (14), it is unclear whether the experimental findings in mice can be translated to a human and/or clinical setting. Ongoing studies are needed to establish the importance of platelet desialylation in ITP.

### C-Reactive Protein and Reactive Oxygen Species

Recently, a role for inflammatory acute-phase protein C-reactive protein (CRP) has also been implied in ITP pathogenesis (216). CRP levels were elevated in ITP patients and enhanced platelet phagocytosis in presence of anti-platelet antibodies *in vitro* and *in vivo*. This effect was ameliorated by IVIg treatment, suggesting that this mechanism may at least in part be mediated *via* FcγRs (216). Phosphorylcholine, a CRP ligand present on cell surfaces, was exposed after antibody-induced oxidative stress. Oxidative stress induced by ITP autoantibodies has also been shown in two separate studies on ITP (217, 218) and appears to be a suitable biomarker for ITP (219). Additionally, the pathophysiological role of reactive oxygen species has long been implied in a model of HIV-initiated ITP (64, 65, 67). In this model, reactive oxygen species induced by platelet antibodies were able to directly lyse platelets, leading to platelet fragmentation. This appears to involve the platelet NADPH pathway and is complement independent (65). Interestingly, treating platelets with dexamethasone was shown to inhibit NADPH oxidase components that partially prevented induction of reactive oxygen species (67). Further studies will be required to elucidate the exact role of CRP, oxidative stress, and autoantibodies or autoreactive CD8^+^ Tc in ITP.

## Model for ITP Pathogenesis

As knowledge on the pathogenesis of ITP develops, definitions become outdated, and lines between primary and secondary ITP are beginning to blur. In other autoimmune diseases, infections are increasingly recognized as one of the primary initiating events that can lead to an autoimmune response. This is not the case for ITP, where it is regarded as a secondary form. However, even in what is called primary ITP, there must be some sort of initiating event that triggers the autoimmune response and exposes platelet antigens. This initiating event will obviously still have consequences for clinical treatment of ITP, whether it is an infection, blood transfusion, drug, or an unknown other trigger. Nonetheless, infections should no longer simply be regarded as a secondary form considering their potential as an initiating event or trigger to expose platelet antigens.

**Figure 3:**
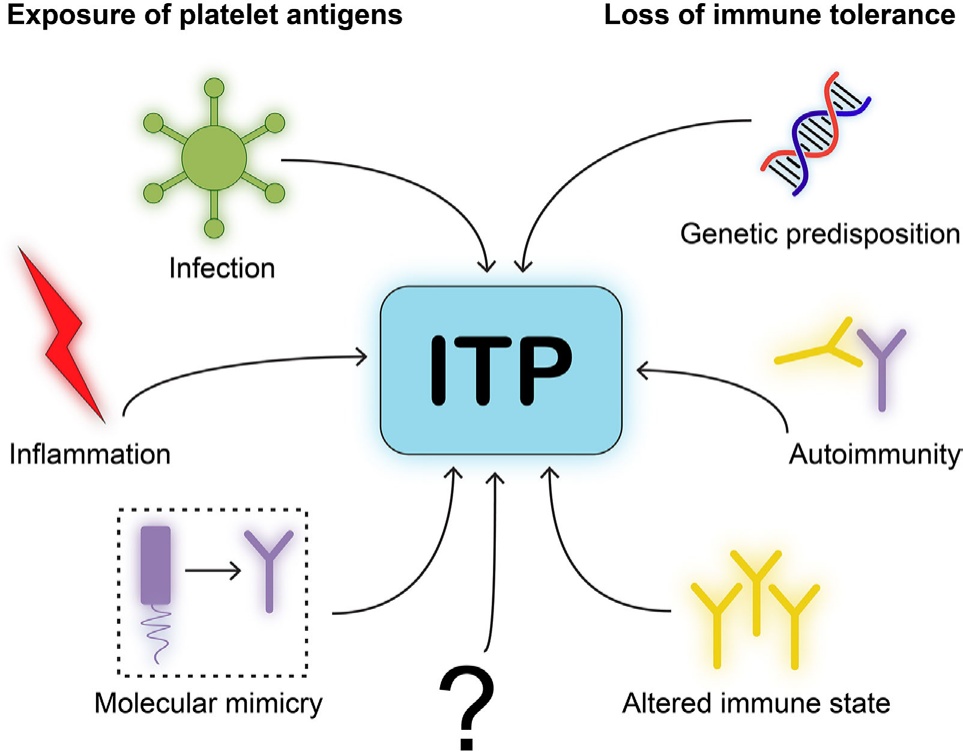
Model for immune thrombocytopenia (ITP) pathogenesis. We postulate a simplified model for the pathogenesis of ITP. One, neo-epitopes on platelet antigens need to be exposed to immune cells. This requires an initiating event or trigger, such as infection, inflammation, or molecular mimicry of viral antigens to resemble platelet glycoproteins. Two, immune cells will need to be able to develop self-reactivity due to loss of immune tolerance. This requires either genetic disposition in immune related-genes, autoimmunity by comorbidities that implies a central dysfunction in tolerance, or an altered immune state such as after organ transplantation.

The number of people developing ITP directly after an infection is small, which suggests that additional factors have to be present during an infection to develop persistent autoimmunity. Individuals with a known autoimmune disease are more prone to develop ITP, indicating that dysregulation of immune homeostasis may contribute to the onset of ITP. Interestingly, most pediatric patients only develop transient thrombocytopenia, which is eventually resolved when the viral antigen is cleared. Meanwhile, similar to other autoimmune disorders, chronic ITP is more prevalent in adult patients, and the incidence increases with age. Based on the currently available data, we propose a simplified model of ITP in which both exposure of platelet antigens and loss of tolerance are required to induce ITP (Figure 3). The specific type of trigger likely determines whether a CD4^+^ T cell-assisted B cell response develops or whether CD8^+^ Tc targeting platelets are induced. Transient forms of ITP may develop if insufficient CD4^+^ T cell help is available for the generation of class-switched, fully affinity matured, strongly binding anti-platelet antibodies. Such antibodies are likely produced by bone marrow-residing plasma cells in a fully developed CD4^+^ T cell-assisted B cell response. We furthermore propose that platelet directed CD8^+^ T cell responses develop following presentation of pathogen-derived peptides on MHC class I that may evoke the formation of CD8^+^ Tc that (cross) react with peptides presented on MHC class I on platelets.

## Future Research

### Emerging Concepts and Opportunities to Unravel the Pathogenesis of ITP

Limited information is available on the autoantigens in ITP and their importance for recognition by immune cells once bound by autoantibodies. Epitopes targeted by platelet autoantibodies seem to differ between patients, coinciding with different responses to therapy and different bleeding phenotypes. The molecular basis for the variable bleeding diathesis in patients with ITP has not yet been fully elucidated. Investigators have primarily made use of ITP sera or plasmas to study the role of autoantibodies. However, these can contain multiple autoantibodies, some potentially undetected by the current methods. Similar to the elegant studied by Roark and co-workers (110), specific autoantibodies should be isolated to further study their effects on platelets, possibly combining characteristics like subclass characterization, epitope specificity, and glycosylation patterns.

### ITP Versus Other Autoimmune Diseases: Lessons to Be Learnt

In other autoimmune disorders such as rheumatoid arthritis, systemic lupus erythematosus, or type 1 diabetes, it has been shown that post-translational modifications of autoantigens can elicit the formation of CD4^+^ T cell responses as well as create neo-epitopes that are recognized by B cells. In view of the common mechanisms involved in loss of tolerance against self, these findings may open novel avenues for dissecting pathways contributing to the onset of ITP.

Evidence has been obtained for post-translational modifications of platelet proteins. Phosphorylation and particularly glycosylation of platelets have been well studied (29, 210, 220, 221), and the importance of platelet glycans is increasingly appreciated. Furthermore, platelets and peripheral blood contain different glycosyltransferases to modify platelet glycans (221), but their relevance in normal platelet physiology is still unclear and their potential relevance for the onset of ITP has not been established. A role for desialylation triggered by platelet autoantibodies or CD8^+^ Tc has been postulated, but it is unknown if this can also lead to the generation of neo-epitopes on the platelet surface (155, 209). Recently, it was also shown that formation of oxidative stress induced neo-epitopes on platelets promotes binding of the acute phase protein CRP resulting in enhanced phagocytosis of IgG-coated platelets (216). It is unclear whether the autoantibodies found in ITP patients are able to recognize such neo-epitopes in similar fashion.

Besides post-translational modifications, platelet membranes are highly dynamic with respect to the expression of cell-surface receptors. GP expression on the platelet surface is tightly regulated by different metalloproteases, such as ADAM10 and ADAM 17 that facilitate receptor shedding (222, 223). Additionally, platelet granules release their content to rapidly increase receptor density on the membrane, such as the well-established activation marker P-selectin (223). These processes are important in both health and disease (224); however, it is unknown if the dynamic shuffling of receptors on the platelet surface is in any way relevant to formation of neo-epitopes in ITP.

The difference between post-translational modifications in other autoimmune diseases and ITP is that most of the modifications mentioned above are induced by autoantibodies in ITP, while modifications in for example the autoantigens that are implicated in rheumatoid arthritis precede the formation of autoantibodies (225–227) and are postulated to be one of the key events that triggers their generation. In fact, infection-induced post-translational modifications of target proteins, such as citrullination of fibrin, are thought to initiate a continuous inflammatory environment, which eventually leads to autoimmunity (225–227). Interestingly, the autoantigens in rheumatoid arthritis are usually located on “static” long-lived cartilage and/or joint proteins, such as fibrin. This is different in ITP, where the autoantigens are located on GPs on platelets that have a limited lifespan. Currently, no information is available with respect to the potential of post-translational modifications of platelet antigens to trigger autoimmune responses. In view of the prominent role of post-translational modifications in the onset of autoimmunity, we speculate that this will provide a novel and interesting avenue for future research to dissect the mechanisms that contribute to the onset of ITP.

## Conclusion

We suggest a simplified model of ITP in which both exposure of platelet antigens and loss of tolerance are required for the onset of ITP, thereby promoting CD4^+^ T cell-assisted B cell responses against platelets. Additionally, we propose that infections resulting in the presentation of pathogen-derived peptides on MHC class I may induce the formation of CD8^+^ Tc that (cross) react with peptides presented on MHC class I on platelets. Specific triggers likely determine the type of autoimmune response against platelets. We speculate that post-translational modifications of platelet antigens harbor potential to generate neo-epitopes that trigger autoimmune responses in ITP, as they do in other autoimmune disorders. Future studies interrogating these hypotheses may yield novel insights into the mechanisms that underlie the development of ITP.

## Author Contributions

MS wrote the manuscript. MR, JV, GV, FL and AJ provided input, made suggestions for improvement, and approved the final version for submission.

## Conflict of Interest Statement

AJ has received travel funding for hematological conferences from Novartis, not specifically for the subject of the manuscript. FL has received an unrestricted grant outside the submitted work from CSL Behring and Shire, and is a consultant for UniQure, NovoNordisk, and Shire, of which the fees go to Erasmus University. JV is a consultant for BioTest. The other authors report no conflict of interest.

## References

[1] Rodeghiero F Stasi R Gernsheimer T Michel M Provan D Arnold DM Standardization of terminology, definitions and outcome criteria in immune thrombocytopenic purpura of adults and children: report from an international working group. Blood (2009) 113(11):2386–93. 10.1182/blood-2008-07-162503 19005182

[2] Provan D Stasi R Newland AC Blanchette VS Bolton-Maggs P Bussel JB International consensus report on the investigation and management of primary immune thrombocytopenia. Blood (2010) 115(2):168–86. 10.1182/blood-2009-06-225565 19846889

[3] Neunert C Lim W Crowther M Cohen A Solberg L Jr Crowther MA The American Society of Hematology 2011 evidence-based practice guideline for immune thrombocytopenia. Blood (2011) 117(16):4190–207. 10.1182/blood-2010-08-302984 21325604

[4] Michel M. Immune thrombocytopenia nomenclature, consensus reports, and guidelines: what are the consequences for daily practice and clinical research? Semin Hematol (2013) 50(Suppl 1):S50–4. 10.1053/j.seminhematol.2013.03.008 23664517

[5] Cines DB Bussel JB Liebman HA Luning Prak ET. The ITP syndrome: pathogenic and clinical diversity. Blood (2009) 113(26):6511–21. 10.1182/blood-2009-01-129155 19395674 PMC2710913

[6] Moulis G Palmaro A Montastruc JL Godeau B Lapeyre-Mestre M Sailler L Epidemiology of incident immune thrombocytopenia: a nationwide population-based study in France. Blood (2014) 124(22):3308–15. 10.1182/blood-2014-05-578336 25305203

[7] Terrell DR Beebe LA Vesely SK Neas BR Segal JB George JN. The incidence of immune thrombocytopenic purpura in children and adults: a critical review of published reports. Am J Hematol (2010) 85(3):174–80. 10.1002/ajh.21616 20131303

[8] Bennett CM Neunert C Grace RF Buchanan G Imbach P Vesely SK Predictors of remission in children with newly diagnosed immune thrombocytopenia: data from the Intercontinental Cooperative ITP Study Group Registry II participants. Pediatr Blood Cancer (2018) 65(1):e26736 10.1002/pbc.26736 28792679

[9] An R Wang PP. Length of stay, hospitalization cost, and in-hospital mortality in US adult inpatients with immune thrombocytopenic purpura, 2006-2012. Vasc Health Risk Manag (2017) 13:15–21. 10.2147/VHRM.S123631 28176930 PMC5268091

[10] Neunert C Noroozi N Norman G Buchanan GR Goy J Nazi I Severe bleeding events in adults and children with primary immune thrombocytopenia: a systematic review. J Thromb Haemost (2015) 13(3):457–64. 10.1111/jth.12813 25495497 PMC4991942

[11] Panzer S Rieger M Vormittag R Eichelberger B Dunkler D Pabinger I. Platelet function to estimate the bleeding risk in autoimmune thrombocytopenia. Eur J Clin Invest (2007) 37(10):814–9. 10.1111/j.1365-2362.2007.01855.x 17727674

[12] Greene LA Chen S Seery C Imahiyerobo AM Bussel JB. Beyond the platelet count: immature platelet fraction and thromboelastometry correlate with bleeding in patients with immune thrombocytopenia. Br J Haematol (2014) 166(4):592–600. 10.1111/bjh.12929 24797389

[13] Frelinger AL III Grace RF Gerrits AJ Berny-Lang MA Brown T Carmichael SL Platelet function tests, independent of platelet count, are associated with bleeding severity in ITP. Blood (2015) 126(7):873–9. 10.1182/blood-2015-02-628461 26138687 PMC4536541

[14] Li R Hoffmeister KM Falet H. Glycans and the platelet life cycle. Platelets (2016) 27(6):505–11. 10.3109/09537104.2016.1171304 27135356 PMC5396182

[15] Shrivastava M. The platelet storage lesion. Transfus Apher Sci (2009) 41(2):105–13. 10.1016/j.transci.2009.07.002 19683964

[16] Devine DV Serrano K. The platelet storage lesion. Clin Lab Med (2010) 30(2):475–87. 10.1016/j.cll.2010.02.002 20513565

[17] Rijkers M van den Eshof BL van der Meer PF van Alphen FPJ de Korte D Leebeek FWG Monitoring storage induced changes in the platelet proteome employing label free quantitative mass spectrometry. Sci Rep (2017) 7(1):11045. 10.1038/s41598-017-11643-w 28887518 PMC5591311

[18] Rijkers M van der Meer PF Bontekoe IJ Daal BB de Korte D Leebeek FW Evaluation of the role of the GPIb-IX-V receptor complex in development of the platelet storage lesion. Vox Sang (2016) 111(3):247–56. 10.1111/vox.12416 27184018

[19] Stegner D vanEeuwijk JMM Angay O Gorelashvili MG Semeniak D Pinnecker J Thrombopoiesis is spatially regulated by the bone marrow vasculature. Nat Commun (2017) 8(1):127. 10.1038/s41467-017-00201-7 28743899 PMC5527048

[20] Italiano JE Jr Lecine P Shivdasani RA Hartwig JH. Blood platelets are assembled principally at the ends of proplatelet processes produced by differentiated megakaryocytes. J Cell Biol (1999) 147(6):1299–312. 10.1083/jcb.147.6.1299 10601342 PMC2168104

[21] Thon JN Montalvo A Patel-Hett S Devine MT Richardson JL Ehrlicher A Cytoskeletal mechanics of proplatelet maturation and platelet release. J Cell Biol (2010) 191(4):861–74. 10.1083/jcb.201006102 21079248 PMC2983072

[22] Lefrancais E Ortiz-Munoz G Caudrillier A Mallavia B Liu F Sayah DM The lung is a site of platelet biogenesis and a reservoir for haematopoietic progenitors. Nature (2017) 544(7648):105–9. 10.1038/nature21706 28329764 PMC5663284

[23] Gurney AL Carver-Moore K de Sauvage FJ Moore MW. Thrombocytopenia in c-mpl-deficient mice. Science (1994) 265(5177):1445–7. 10.1126/science.8073287 8073287

[24] Alexander WS Roberts AW Nicola NA Li R Metcalf D. Deficiencies in progenitor cells of multiple hematopoietic lineages and defective megakaryocytopoiesis in mice lacking the thrombopoietic receptor c-Mpl. Blood (1996) 87(6):2162–70. 8630375

[25] Varghese LN Defour JP Pecquet C Constantinescu SN The thrombopoietin receptor: structural basis of traffic and activation by ligand, mutations, agonists, and mutated calreticulin. Front Endocrinol (2017) 8:59 10.3389/fendo.2017.00059 PMC5374145 28408900

[26] de Sauvage FJ Carver-Moore K Luoh SM Ryan A Dowd M Eaton DL Physiological regulation of early and late stages of megakaryocytopoiesis by thrombopoietin. J Exp Med (1996) 183(2):651–6. 10.1084/jem.183.2.651 8627177 PMC2192470

[27] Grozovsky R Begonja AJ Liu K Visner G Hartwig JH Falet H The Ashwell-Morell receptor regulates hepatic thrombopoietin production via JAK2-STAT3 signaling. Nat Med (2015) 21(1):47–54. 10.1038/nm.3770 25485912 PMC4303234

[28] Mason KD Carpinelli MR Fletcher JI Collinge JE Hilton AA Ellis S Programmed anuclear cell death delimits platelet life span. Cell (2007) 128(6):1173–86. 10.1016/j.cell.2007.01.037 17382885

[29] Grozovsky R Giannini S Falet H Hoffmeister KM. Regulating billions of blood platelets: glycans and beyond. Blood (2015) 126(16):1877–84. 10.1182/blood-2015-01-569129 26330242 PMC4608239

[30] Tahara T Usuki K Sato H Ohashi H Morita H Tsumura H A sensitive sandwich ELISA for measuring thrombopoietin in human serum: serum thrombopoietin levels in healthy volunteers and in patients with haemopoietic disorders. Br J Haematol (1996) 93(4):783–8. 10.1046/j.1365-2141.1996.d01-1741.x 8703803

[31] Kosugi S Kurata Y Tomiyama Y Tahara T Kato T Tadokoro S Circulating thrombopoietin level in chronic immune thrombocytopenic purpura. Br J Haematol (1996) 93(3):704–6. 10.1046/j.1365-2141.1996.d01-1702.x 8652398

[32] Rodeghiero F Carli G. Beyond immune thrombocytopenia: the evolving role of thrombopoietin receptor agonists. Ann Hematol (2017) 96(9):1421–34. 10.1007/s00277-017-2953-6 28275823

[33] Wu KH Peng CT Li TC Wan L Tsai CH Lan SJ Interleukin 4, interleukin 6 and interleukin 10 polymorphisms in children with acute and chronic immune thrombocytopenic purpura. Br J Haematol (2005) 128(6):849–52. 10.1111/j.1365-2141.2005.05385.x 15755291

[34] Emmerich F Bal G Barakat A Milz J Muhle C Martinez-Gamboa L High-level serum B-cell activating factor and promoter polymorphisms in patients with idiopathic thrombocytopenic purpura. Br J Haematol (2007) 136(2):309–14. 10.1111/j.1365-2141.2006.06431.x 17156395

[35] Rocha AM De Souza C Rocha GA De Melo FF Saraiva IS Clementino NC IL1RN VNTR and IL2-330 polymorphic genes are independently associated with chronic immune thrombocytopenia. Br J Haematol (2010) 150(6):679–84. 10.1111/j.1365-2141.2010.08318.x 20626741

[36] Pehlivan M Okan V Sever T Balci SO Yilmaz M Babacan T Investigation of TNF-alpha, TGF-beta 1, IL-10, IL-6, IFN-gamma, MBL, GPIA, and IL1A gene polymorphisms in patients with idiopathic thrombocytopenic purpura. Platelets (2011) 22(8):588–95. 10.3109/09537104.2011.577255 21591983

[37] Saitoh T Tsukamoto N Koiso H Mitsui T Yokohama A Handa H Interleukin-17F gene polymorphism in patients with chronic immune thrombocytopenia. Eur J Haematol (2011) 87(3):253–8. 10.1111/j.1600-0609.2011.01651.x 21615796

[38] Kuwana M Kaburaki J Pandey JP Murata M Kawakami Y Inoko H HLA class II alleles in Japanese patients with immune thrombocytopenic purpura. Associations with anti-platelet glycoprotein autoantibodies and responses to splenectomy. Tissue Antigens (2000) 56(4):337–43. 10.1034/j.1399-0039.2000.560405.x 11098933

[39] Leung AY Hawkins BR Chim CS Kwong YY Liang RH Genetic analysis of HLA-typing in Chinese patients with idiopathic thrombocytopenic purpura. Haematologica (2001) 86(2):221–2. 11224501

[40] Stanworth SJ Turner DM Brown J McCloskey D Brown C Provan D Major histocompatibility complex susceptibility genes and immune thrombocytopenic purpura in Caucasian adults. Hematology (2002) 7(2):119–21. 10.1080/10245330290028605 12186703

[41] El Neanaey WA Barakat SS Ahmed MA El Nabie WM Ahmed ME. The relation between HLA-DRB1 alleles and the outcome of therapy in children with idiopathic thrombocytopenic purpura. Egypt J Immunol (2005) 12(2):29–38. 17977208

[42] Hopkins LM Davis JM Buchli R Vangundy RS Schwartz KA Gerlach JA MHC class I-associated peptides identified from normal platelets and from individuals with idiopathic thrombocytopenic purpura. Hum Immunol (2005) 66(8):874–83. 10.1016/j.humimm.2005.06.004 16216671

[43] Maia MH Peixoto Rde L de Lima CP Magalhaes M Sena L Costa Pdo S Predisposition to idiopathic thrombocytopenic purpura maps close to the major histocompatibility complex class I chain-related gene A. Hum Immunol (2009) 70(3):179–83. 10.1016/j.humimm.2009.01.011 19280715

[44] Negi RR Bhoria P Pahuja A Saikia B Varma N Malhotra P Investigation of the possible association between the HLA antigens and idiopathic thrombocytopenic purpura (ITP). Immunol Invest (2012) 41(2):117–28. 10.3109/08820139.2011.593218 21806423

[45] Ho WL Lu MY Hu FC Lee CC Huang LM Jou ST Clinical features and major histocompatibility complex genes as potential susceptibility factors in pediatric immune thrombocytopenia. J Formos Med Assoc (2012) 111(7):370–9. 10.1016/j.jfma.2011.06.025 22817814

[46] Jernas M Hou Y Stromberg Celind F Shao L Nookaew I Wang Q Differences in gene expression and cytokine levels between newly diagnosed and chronic pediatric ITP. Blood (2013) 122(10):1789–92. 10.1182/blood-2013-05-502807 23869085

[47] Foster CB Zhu S Erichsen HC Lehrnbecher T Hart ES Choi E Polymorphisms in inflammatory cytokines and Fcgamma receptors in childhood chronic immune thrombocytopenic purpura: a pilot study. Br J Haematol (2001) 113(3):596–9. 10.1046/j.1365-2141.2001.02807.x 11380443

[48] Carcao MD Blanchette VS Wakefield CD Stephens D Ellis J Matheson K Fcgamma receptor IIa and IIIa polymorphisms in childhood immune thrombocytopenic purpura. Br J Haematol (2003) 120(1):135–41. 10.1046/j.1365-2141.2003.04033.x 12492589

[49] Bruin M Bierings M Uiterwaal C Revesz T Bode L Wiesman ME Platelet count, previous infection and FCGR2B genotype predict development of chronic disease in newly diagnosed idiopathic thrombocytopenia in childhood: results of a prospective study. Br J Haematol (2004) 127(5):561–7. 10.1111/j.1365-2141.2004.05235.x 15566359

[50] Breunis WB van Mirre E Bruin M Geissler J de Boer M Peters M Copy number variation of the activating FCGR2C gene predisposes to idiopathic thrombocytopenic purpura. Blood (2008) 111(3):1029–38. 10.1182/blood-2007-03-079913 17827395

[51] Amorim DM Silveira Vda S Scrideli CA Queiroz RG Tone LG Fcgamma receptor gene polymorphisms in childhood immune thrombocytopenic purpura. J Pediatr Hematol Oncol (2012) 34(5):349–52. 10.1097/MPH.0b013e3182580908 22713706

[52] Eyada TK Farawela HM Khorshied MM Shaheen IA Selim NM Khalifa IA FcgammaRIIa and FcgammaRIIIa genetic polymorphisms in a group of pediatric immune thrombocytopenic purpura in Egypt. Blood Coagul Fibrinolysis (2012) 23(1):64–8. 10.1097/MBC.0b013e32834ddf2f 22123287

[53] Papagianni A Economou M Tragiannidis A Karatza E Samarah F Gombakis N FcgammaRIIa and FcgammaRIIIa polymorphisms in childhood primary immune thrombocytopenia: implications for disease pathogenesis and outcome. Blood Coagul Fibrinolysis (2013) 24(1):35–9. 10.1097/MBC.0b013e328359bc3b 23249566

[54] Wang D Hu SL Cheng XL Yang JY. FCGR2A rs1801274 polymorphism is associated with risk of childhood-onset idiopathic (immune) thrombocytopenic purpura: evidence from a meta-analysis. Thromb Res (2014) 134(6):1323–7. 10.1016/j.thromres.2014.10.003 25457587

[55] Simeoni I Stephens JC Hu F Deevi SV Megy K Bariana TK A high-throughput sequencing test for diagnosing inherited bleeding, thrombotic, and platelet disorders. Blood (2016) 127(23):2791–803. 10.1182/blood-2015-12-688267 27084890 PMC5016734

[56] Bariana TK Ouwehand WH Guerrero JA Gomez K BRIDGE Bleeding, Thrombotic and Platelet Disorders and ThromboGenomics Consortia. Dawning of the age of genomics for platelet granule disorders: improving insight, diagnosis and management. Br J Haematol (2017) 176(5):705–20. 10.1111/bjh.14471 27984638

[57] Takahashi T Yujiri T Shinohara K Inoue Y Sato Y Fujii Y Molecular mimicry by *Helicobacter pylori* CagA protein may be involved in the pathogenesis of *H. pylori*-associated chronic idiopathic thrombocytopenic purpura. Br J Haematol (2004) 124(1):91–6. 10.1046/j.1365-2141.2003.04735.x 14675413

[58] Stasi R Sarpatwari A Segal JB Osborn J Evangelista ML Cooper N Effects of eradication of *Helicobacter pylori* infection in patients with immune thrombocytopenic purpura: a systematic review. Blood (2009) 113(6):1231–40. 10.1182/blood-2008-07-167155 18945961

[59] Rajan SK Espina BM Liebman HA. Hepatitis C virus-related thrombocytopenia: clinical and laboratory characteristics compared with chronic immune thrombocytopenic purpura. Br J Haematol (2005) 129(6):818–24. 10.1111/j.1365-2141.2005.05542.x 15953010

[60] Zhang W Nardi MA Borkowsky W Li Z Karpatkin S. Role of molecular mimicry of hepatitis C virus protein with platelet GPIIIa in hepatitis C-related immunologic thrombocytopenia. Blood (2009) 113(17):4086–93. 10.1182/blood-2008-09-181073 19023115 PMC2673130

[61] Hohmann AW Booth K Peters V Gordon DL Comacchio RM. Common epitope on HIV p24 and human platelets. Lancet (1993) 342(8882):1274–5. 10.1016/0140-6736(93)92363-X 7694021

[62] Bettaieb A Oksenhendler E Duedari N Bierling P. Cross-reactive antibodies between HIV-gp120 and platelet gpIIIa (CD61) in HIV-related immune thrombocytopenic purpura. Clin Exp Immunol (1996) 103(1):19–23. 10.1046/j.1365-2249.1996.917606.x 8565280 PMC2200313

[63] Dominguez V Gevorkian G Govezensky T Rodriguez H Viveros M Cocho G Antigenic homology of HIV-1 GP41 and human platelet glycoprotein GPIIIa (integrin beta3). J Acquir Immune Defic Syndr Hum Retrovirol (1998) 17(5):385–90. 10.1097/00042560-199804150-00001 9562039

[64] Nardi M Tomlinson S Greco MA Karpatkin S. Complement-independent, peroxide-induced antibody lysis of platelets in HIV-1-related immune thrombocytopenia. Cell (2001) 106(5):551–61. 10.1016/S0092-8674(01)00477-9 11551503

[65] Nardi M Feinmark SJ Hu L Li Z Karpatkin S. Complement-independent Ab-induced peroxide lysis of platelets requires 12-lipoxygenase and a platelet NADPH oxidase pathway. J Clin Invest (2004) 113(7):973–80. 10.1172/JCI20726 15057303 PMC379327

[66] Li Z Nardi MA Karpatkin S. Role of molecular mimicry to HIV-1 peptides in HIV-1-related immunologic thrombocytopenia. Blood (2005) 106(2):572–6. 10.1182/blood-2005-01-0243 15774614 PMC1895171

[67] Nardi MA Gor Y Feinmark SJ Xu F Karpatkin S Platelet particle formation by anti GPIIIa49-66 Ab, Ca2+ ionophore A23187, and phorbol myristate acetate is induced by reactive oxygen species and inhibited by dexamethasone blockade of platelet phospholipase A2, 12-lipoxygenase, and NADPH oxidase. Blood (2007) 110(6):1989–96. 10.1182/blood-2006-10-054064 17545506 PMC1976358

[68] DiMaggio D Anderson A Bussel JB. Cytomegalovirus can make immune thrombocytopenic purpura refractory. Br J Haematol (2009) 146(1):104–12. 10.1111/j.1365-2141.2009.07714.x 19438507

[69] Wu Z Zhou J Wei X Wang X Li Y Peng B The role of Epstein-Barr virus (EBV) and cytomegalovirus (CMV) in immune thrombocytopenia. Hematology (2013) 18(5):295–9. 10.1179/1607845413Y.0000000084 23540727

[70] Wright JF Blanchette VS Wang H Arya N Petric M Semple JW Characterization of platelet-reactive antibodies in children with varicella-associated acute immune thrombocytopenic purpura (ITP). Br J Haematol (1996) 95(1):145–52. 10.1046/j.1365-2141.1996.d01-1872.x 8857953

[71] Musaji A Cormont F Thirion G Cambiaso CL Coutelier JP. Exacerbation of autoantibody-mediated thrombocytopenic purpura by infection with mouse viruses. Blood (2004) 104(7):2102–6. 10.1182/blood-2004-01-0310 15205264

[72] Mantadakis E Farmaki E Buchanan GR. Thrombocytopenic purpura after measles-mumps-rubella vaccination: a systematic review of the literature and guidance for management. J Pediatr (2010) 156(4):623–8. 10.1016/j.jpeds.2009.10.015 20097358

[73] Grimaldi-Bensouda L Michel M Aubrun E Leighton P Viallard JF Adoue D A case-control study to assess the risk of immune thrombocytopenia associated with vaccines. Blood (2012) 120(25):4938–44. 10.1182/blood-2012-05-431098 23100310

[74] Rose NR. Negative selection, epitope mimicry and autoimmunity. Curr Opin Immunol (2017) 49:51–5. 10.1016/j.coi.2017.08.014 29102863

[75] Aslam R Speck ER Kim M Crow AR Bang KW Nestel FP Platelet toll-like receptor expression modulates lipopolysaccharide-induced thrombocytopenia and tumor necrosis factor-alpha production in vivo. Blood (2006) 107(2):637–41. 10.1182/blood-2005-06-2202 16179373

[76] Aster RH Bougie DW Drug-induced immune thrombocytopenia. N Engl J Med (2007) 357(6):580–7. 10.1056/NEJMra066469 17687133

[77] Cines DB Liebman H Stasi R. Pathobiology of secondary immune thrombocytopenia. Semin Hematol (2009) 46(1 Suppl 2):S2–14. 10.1053/j.seminhematol.2008.12.005 19245930 PMC2682438

[78] van Leeuwen EF van der Ven JT Engelfriet CP von dem Borne AE. Specificity of autoantibodies in autoimmune thrombocytopenia. Blood (1982) 59(1):23–6. 7032627

[79] McMillan R Tani P Millard F Berchtold P Renshaw L Woods VL Jr. Platelet-associated and plasma anti-glycoprotein autoantibodies in chronic ITP. Blood (1987) 70(4):1040–5. 3651598

[80] He R Reid DM Jones CE Shulman NR. Spectrum of Ig classes, specificities, and titers of serum antiglycoproteins in chronic idiopathic thrombocytopenic purpura. Blood (1994) 83(4):1024–32. 8111044

[81] Kiyomizu K Kashiwagi H Nakazawa T Tadokoro S Honda S Kanakura Y Recognition of highly restricted regions in the beta-propeller domain of alphaIIb by platelet-associated anti-alphaIIbbeta3 autoantibodies in primary immune thrombocytopenia. Blood (2012) 120(7):1499–509. 10.1182/blood-2012-02-409995 22730538

[82] Beer JH Rabaglio M Berchtold P von Felten A Clemetson KJ Tsakiris DA Autoantibodies against the platelet glycoproteins (GP) IIb/IIIa, Ia/IIa, and IV and partial deficiency in GPIV in a patient with a bleeding disorder and a defective platelet collagen interaction. Blood (1993) 82(3):820–9. 7687896

[83] Boylan B Chen H Rathore V Paddock C Salacz M Friedman KD Anti-GPVI-associated ITP: an acquired platelet disorder caused by autoantibody-mediated clearance of the GPVI/FcRgamma-chain complex from the human platelet surface. Blood (2004) 104(5):1350–5. 10.1182/blood-2004-03-0896 15150079

[84] Najean Y Rain JD Billotey C. The site of destruction of autologous 111In-labelled platelets and the efficiency of splenectomy in children and adults with idiopathic thrombocytopenic purpura: a study of 578 patients with 268 splenectomies. Br J Haematol (1997) 97(3):547–50. 10.1046/j.1365-2141.1997.832723.x 9207397

[85] Kuwana M Okazaki Y Kaburaki J Kawakami Y Ikeda Y. Spleen is a primary site for activation of platelet-reactive T and B cells in patients with immune thrombocytopenic purpura. J Immunol (2002) 168(7):3675–82. 10.4049/jimmunol.168.7.3675 11907134

[86] Catani L Fagioli ME Tazzari PL Ricci F Curti A Rovito M Dendritic cells of immune thrombocytopenic purpura (ITP) show increased capacity to present apoptotic platelets to T lymphocytes. Exp Hematol (2006) 34(7):879–87. 10.1016/j.exphem.2006.03.009 16797415

[87] Kuwana M Okazaki Y Ikeda Y. Splenic macrophages maintain the anti-platelet autoimmune response via uptake of opsonized platelets in patients with immune thrombocytopenic purpura. J Thromb Haemost (2009) 7(2):322–9. 10.1111/j.1538-7836.2008.03161.x 18826388

[88] Chang M Nakagawa PA Williams SA Schwartz MR Imfeld KL Buzby JS Immune thrombocytopenic purpura (ITP) plasma and purified ITP monoclonal autoantibodies inhibit megakaryocytopoiesis in vitro. Blood (2003) 102(3):887–95. 10.1182/blood-2002-05-1475 12676790

[89] McMillan R Wang L Tomer A Nichol J Pistillo J. Suppression of in vitro megakaryocyte production by antiplatelet autoantibodies from adult patients with chronic ITP. Blood (2004) 103(4):1364–9. 10.1182/blood-2003-08-2672 14576051

[90] Lev PR Grodzielski M Goette NP Glembotsky AC Espasandin YR Pierdominici MS Impaired proplatelet formation in immune thrombocytopenia: a novel mechanism contributing to decreased platelet count. Br J Haematol (2014) 165(6):854–64. 10.1111/bjh.12832 24673454

[91] Tsubakio T Tani P Curd JG McMillan R Complement activation in vitro by antiplatelet antibodies in chronic immune thrombocytopenic purpura. Br J Haematol (1986) 63(2):293–300. 10.1111/j.1365-2141.1986.tb05552.x 3718872

[92] Peerschke EI Andemariam B Yin W Bussel JB. Complement activation on platelets correlates with a decrease in circulating immature platelets in patients with immune thrombocytopenic purpura. Br J Haematol (2010) 148(4):638–45. 10.1111/j.1365-2141.2009.07995.x 19925495 PMC5004348

[93] Najaoui A Bakchoul T Stoy J Bein G Rummel MJ Santoso S Autoantibody-mediated complement activation on platelets is a common finding in patients with immune thrombocytopenic purpura (ITP). Eur J Haematol (2012) 88(2):167–74. 10.1111/j.1600-0609.2011.01718.x 21985182

[94] Goette NP Glembotsky AC Lev PR Grodzielski M Contrufo G Pierdominici MS Platelet apoptosis in adult immune thrombocytopenia: insights into the mechanism of damage triggered by auto-antibodies. PLoS One (2016) 11(8):e0160563. 10.1371/journal.pone.0160563 27494140 PMC4975454

[95] George JN Platelet immunoglobulin G: its significance for the evaluation of thrombocytopenia and for understanding the origin of alpha-granule proteins. Blood (1990) 76(5):859–70. 2203482

[96] Nagelkerke SQ Kuijpers TW Immunomodulation by IVIg and the role of Fc-gamma receptors: classic mechanisms of action after all? Front Immunol (2014) 5:674 10.3389/fimmu.2014.00674 25653650 PMC4301001

[97] Kiefel V Santoso S Weisheit M Mueller-Eckhardt C Monoclonal antibody-specific immobilization of platelet antigens (MAIPA): a new tool for the identification of platelet-reactive antibodies. Blood (1987) 70(6):1722–6. 2445398

[98] Fujisawa K Tani P O’Toole TE Ginsberg MH McMillan R. Different specificities of platelet-associated and plasma autoantibodies to platelet GPIIb-IIIa in patients with chronic immune thrombocytopenic purpura. Blood (1992) 79(6):1441–6. 1547343

[99] Crossley A Calvert JE Taylor PR Dickinson AM A comparison of monoclonal antibody immobilization of platelet antigen (MAIPA) and immunobead methods for detection of GPIIb/IIIa antiplatelet antibodies in immune thrombocytopenic purpura. Transfus Med (1997) 7(2):127–34. 10.1046/j.1365-3148.1997.d01-15.x 9195699

[100] Cines DB Wilson SB Tomaski A Schreiber AD. Platelet antibodies of the IgM class in immune thrombocytopenic purpura. J Clin Invest (1985) 75(4):1183–90. 10.1172/JCI111814 4039335 PMC425443

[101] Winiarski J. IgG and IgM antibodies to platelet membrane glycoprotein antigens in acute childhood idiopathic thrombocytopenic purpura. Br J Haematol (1989) 73(1):88–92. 10.1111/j.1365-2141.1989.tb00225.x 2803983

[102] Nishioka T Yamane T Takubo T Ohta K Park K Hino M. Detection of various platelet-associated immunoglobulins by flow cytometry in idiopathic thrombocytopenic purpura. Cytometry B Clin Cytom (2005) 68(1):37–42. 10.1002/cyto.b.20067 16184616

[103] Chan H Moore JC Finch CN Warkentin TE Kelton JG. The IgG subclasses of platelet-associated autoantibodies directed against platelet glycoproteins IIb/IIIa in patients with idiopathic thrombocytopenic purpura. Br J Haematol (2003) 122(5):818–24. 10.1046/j.1365-2141.2003.04509.x 12930395

[104] Hymes K Schur PH Karpatkin S. Heavy-chain subclass of round antiplatelet IgG in autoimmune thrombocytopenic purpura. Blood (1980) 56(1):84–7. 6770931

[105] Vidarsson G Dekkers G Rispens T. IgG subclasses and allotypes: from structure to effector functions. Front Immunol (2014) 5:520. 10.3389/fimmu.2014.00520 25368619 PMC4202688

[106] Sonneveld ME Natunen S Sainio S Koeleman CA Holst S Dekkers G Glycosylation pattern of anti-platelet IgG is stable during pregnancy and predicts clinical outcome in alloimmune thrombocytopenia. Br J Haematol (2016) 174(2):310–20. 10.1111/bjh.14053 27017954

[107] Sonneveld ME de Haas M Koeleman C de Haan N Zeerleder SS Ligthart PC Patients with IgG1-anti-red blood cell autoantibodies show aberrant Fc-glycosylation. Sci Rep (2017) 7(1):8187. 10.1038/s41598-017-08654-y 28811589 PMC5557851

[108] Christie DJ Sauro SC Fairbanks KD Kay NE. Detection of clonal platelet antibodies in immunologically-mediated thrombocytopenias: association with circulating clonal/oligoclonal B cells. Br J Haematol (1993) 85(2):277–84. 10.1111/j.1365-2141.1993.tb03167.x 8280601

[109] Kim J Park CJ Chi HS Kim MJ Seo JJ Moon HN Idiopathic thrombocytopenic purpura: better therapeutic responses of patients with B- or T-cell clonality than patients without clonality. Int J Hematol (2003) 78(5):461–6. 10.1007/BF02983822 14704042

[110] Roark JH Bussel JB Cines DB Siegel DL. Genetic analysis of autoantibodies in idiopathic thrombocytopenic purpura reveals evidence of clonal expansion and somatic mutation. Blood (2002) 100(4):1388–98. 12149222

[111] Stockelberg D Hou M Jacobsson S Kutti J Wadenvik H. Evidence for a light chain restriction of glycoprotein Ib/IX and IIb/IIIa reactive antibodies in chronic idiopathic thrombocytopenic purpura (ITP). Br J Haematol (1995) 90(1):175–9. 10.1111/j.1365-2141.1995.tb03397.x 7786782

[112] Stockelberg D Hou M Jacobsson S Kutti J Wadenvik H. Light chain-restricted autoantibodies in chronic idiopathic thrombocytopenic purpura, but no evidence for circulating clone B-lymphocytes. Ann Hematol (1996) 72(1):29–34. 10.1007/BF00663013 8605277

[113] van der Harst D de Jong D Limpens J Kluin PM Rozier Y van Ommen GJ Clonal B-cell populations in patients with idiopathic thrombocytopenic purpura. Blood (1990) 76(11):2321–6. 1701666

[114] Veneri D De Matteis G Solero P Federici F Zanuso C Guizzardi E Analysis of B- and T-cell clonality and HLA class II alleles in patients with idiopathic thrombocytopenic purpura: correlation with *Helicobacter pylori* infection and response to eradication treatment. Platelets (2005) 16(5):307–11. 10.1080/09537100400028685 16011982

[115] Voelkerding KV Sandhaus LM Belov L Frenkel L Ettinger LJ Raska K Jr. Clonal B-cell proliferation in an infant with congenital HIV infection and immune thrombocytopenia. Am J Clin Pathol (1988) 90(4):470–4. 10.1093/ajcp/90.4.470 3263038

[116] Fujisawa K O’Toole TE Tani P Loftus JC Plow EF Ginsberg MH Autoantibodies to the presumptive cytoplasmic domain of platelet glycoprotein IIIa in patients with chronic immune thrombocytopenic purpura. Blood (1991) 77(10):2207–13. 1709376

[117] Kosugi S Tomiyama Y Honda S Kato H Kiyoi T Kashiwagi H Platelet-associated anti-GPIIb-IIIa autoantibodies in chronic immune thrombocytopenic purpura recognizing epitopes close to the ligand-binding site of glycoprotein (GP) IIb. Blood (2001) 98(6):1819–27. 10.1182/blood.V98.6.1819 11535516

[118] McMillan R Lopez-Dee J Loftus JC. Autoantibodies to alpha(IIb)beta(3) in patients with chronic immune thrombocytopenic purpura bind primarily to epitopes on alpha(IIb). Blood (2001) 97(7):2171–2. 10.1182/blood.V97.7.2171 11264188

[119] Kekomaki R Dawson B McFarland J Kunicki TJ. Localization of human platelet autoantigens to the cysteine-rich region of glycoprotein IIIa. J Clin Invest (1991) 88(3):847–54. 10.1172/JCI115386 1715887 PMC295471

[120] McMillan R Wang L Lopez-Dee J Jiu S Loftus JC. Many alphaIIbbeta3 autoepitopes in chronic immune thrombocytopenic purpura are localized to alphaIIb between amino acids L1 and Q459. Br J Haematol (2002) 118(4):1132–6. 10.1046/j.1365-2141.2002.03751.x 12199797

[121] Kosugi S Tomiyama Y Honda S Kashiwagi H Shiraga M Tadokoro S Anti-alphavbeta3 antibodies in chronic immune thrombocytopenic purpura. Thromb Haemost (2001) 85(1):36–41. 10.1055/s-0037-1612660 11204584

[122] Berchtold P Wenger M. Autoantibodies against platelet glycoproteins in autoimmune thrombocytopenic purpura: their clinical significance and response to treatment. Blood (1993) 81(5):1246–50. 8443385

[123] Zeng Q Zhu L Tao L Bao J Yang M Simpson EK Relative efficacy of steroid therapy in immune thrombocytopenia mediated by anti-platelet GPIIbIIIa versus GPIbalpha antibodies. Am J Hematol (2012) 87(2):206–8. 10.1002/ajh.22211 22139961

[124] Peng J Ma SH Liu J Hou Y Liu XM Niu T Association of autoantibody specificity and response to intravenous immunoglobulin G therapy in immune thrombocytopenia: a multicenter cohort study. J Thromb Haemost (2014) 12(4):497–504. 10.1111/jth.12524 24517219

[125] Semple JW Freedman J. Increased antiplatelet T helper lymphocyte reactivity in patients with autoimmune thrombocytopenia. Blood (1991) 78(10):2619–25. 1840468

[126] Semple JW Milev Y Cosgrave D Mody M Hornstein A Blanchette V Differences in serum cytokine levels in acute and chronic autoimmune thrombocytopenic purpura: relationship to platelet phenotype and antiplatelet T-cell reactivity. Blood (1996) 87(10):4245–54. 8639783

[127] Ogawara H Handa H Morita K Hayakawa M Kojima J Amagai H High Th1/Th2 ratio in patients with chronic idiopathic thrombocytopenic purpura. Eur J Haematol (2003) 71(4):283–8. 10.1034/j.1600-0609.2003.00138.x 12950238

[128] Panitsas FP Mouzaki A. Effect of splenectomy on type-1/type-2 cytokine gene expression in a patient with adult idiopathic thrombocytopenic purpura (ITP). BMC Blood Disord (2004) 4(1):4. 10.1186/1471-2326-4-4 15491502 PMC524488

[129] Wang T Zhao H Ren H Guo J Xu M Yang R Type 1 and type 2 T-cell profiles in idiopathic thrombocytopenic purpura. Haematologica (2005) 90(7):914–23. 15996929

[130] Stasi R Del Poeta G Stipa E Evangelista ML Trawinska MM Cooper N Response to B-cell depleting therapy with rituximab reverts the abnormalities of T-cell subsets in patients with idiopathic thrombocytopenic purpura. Blood (2007) 110(8):2924–30. 10.1182/blood-2007-02-068999 17548576

[131] Kuwana M Kaburaki J Ikeda Y Autoreactive T cells to platelet GPIIb-IIIa in immune thrombocytopenic purpura. Role in production of anti-platelet autoantibody. J Clin Invest (1998) 102(7):1393–402. 10.1172/JCI4238 9769332 PMC508987

[132] Kuwana M Kaburaki J Kitasato H Kato M Kawai S Kawakami Y Immunodominant epitopes on glycoprotein IIb-IIIa recognized by autoreactive T cells in patients with immune thrombocytopenic purpura. Blood (2001) 98(1):130–9. 10.1182/blood.V98.1.130 11418472

[133] Chen X Oppenheim JJ. Th17 cells and Tregs: unlikely allies. J Leukoc Biol (2014) 95(5):723–31. 10.1189/jlb.1213633 24563509 PMC3984971

[134] Ma D Zhu X Zhao P Zhao C Li X Zhu Y Profile of Th17 cytokines (IL-17, TGF-beta, IL-6) and Th1 cytokine (IFN-gamma) in patients with immune thrombocytopenic purpura. Ann Hematol (2008) 87(11):899–904. 10.1007/s00277-008-0535-3 18600325

[135] Guo ZX Chen ZP Zheng CL Jia HR Ge J Gu DS The role of Th17 cells in adult patients with chronic idiopathic thrombocytopenic purpura. Eur J Haematol (2009) 82(6):488–9. 10.1111/j.1600-0609.2009.01229.x 19187277

[136] Sollazzo D Trabanelli S Curti A Vianelli N Lemoli RM Catani L Circulating CD4+CD161+CD196+ Th17 cells are not increased in immune thrombocytopenia. Haematologica (2011) 96(4):632–4. 10.3324/haematol.2010.038638 21357705 PMC3069245

[137] Zhang J Ma D Zhu X Qu X Ji C Hou M Elevated profile of Th17, Th1 and Tc1 cells in patients with immune thrombocytopenic purpura. Haematologica (2009) 94(9):1326–9. 10.3324/haematol.2009.007823 19734430 PMC2738732

[138] Zhu X Ma D Zhang J Peng J Qu X Ji C Elevated interleukin-21 correlated to Th17 and Th1 cells in patients with immune thrombocytopenia. J Clin Immunol (2010) 30(2):253–9. 10.1007/s10875-009-9353-1 19997967

[139] Rocha AM Souza C Rocha GA de Melo FF Clementino NC Marino MC The levels of IL-17A and of the cytokines involved in Th17 cell commitment are increased in patients with chronic immune thrombocytopenia. Haematologica (2011) 96(10):1560–4. 10.3324/haematol.2011.046417 21972211 PMC3186321

[140] Hu Y Ma DX Shan NN Zhu YY Liu XG Zhang L Increased number of Tc17 and correlation with Th17 cells in patients with immune thrombocytopenia. PLoS One (2011) 6(10):e26522. 10.1371/journal.pone.0026522 22039505 PMC3200336

[141] Ji L Zhan Y Hua F Li F Zou S Wang W The ratio of Treg/Th17 cells correlates with the disease activity of primary immune thrombocytopenia. PLoS One (2012) 7(12):e50909. 10.1371/journal.pone.0050909 23226546 PMC3513316

[142] Eyerich K Dimartino V Cavani A. IL-17 and IL-22 in immunity: driving protection and pathology. Eur J Immunol (2017) 47(4):607–14. 10.1002/eji.201646723 28295238

[143] Eyerich S Eyerich K Cavani A Schmidt-Weber C IL-17 and IL-22: siblings, not twins. Trends Immunol (2010) 31(9):354–61. 10.1016/j.it.2010.06.004 20691634

[144] Eyerich S Eyerich K Pennino D Carbone T Nasorri F Pallotta S Th22 cells represent a distinct human T cell subset involved in epidermal immunity and remodeling. J Clin Invest (2009) 119(12):3573–85. 10.1172/JCI40202 19920355 PMC2786807

[145] Cao J Chen C Zeng L Li L Li X Li Z Elevated plasma IL-22 levels correlated with Th1 and Th22 cells in patients with immune thrombocytopenia. Clin Immunol (2011) 141(1):121–3. 10.1016/j.clim.2011.05.003 21652269

[146] Hu Y Li H Zhang L Shan B Xu X Li H Elevated profiles of Th22 cells and correlations with Th17 cells in patients with immune thrombocytopenia. Hum Immunol (2012) 73(6):629–35. 10.1016/j.humimm.2012.04.015 22537755

[147] Audia S Rossato M Santegoets K Spijkers S Wichers C Bekker C Splenic TFH expansion participates in B-cell differentiation and antiplatelet-antibody production during immune thrombocytopenia. Blood (2014) 124(18):2858–66. 10.1182/blood-2014-03-563445 25232056

[148] Olsson B Andersson PO Jernas M Jacobsson S Carlsson B Carlsson LM T-cell-mediated cytotoxicity toward platelets in chronic idiopathic thrombocytopenic purpura. Nat Med (2003) 9(9):1123–4. 10.1038/nm921 12937414

[149] Ware RE Howard TA. Phenotypic and clonal analysis of T lymphocytes in childhood immune thrombocytopenic purpura. Blood (1993) 82(7):2137–42. 8400263

[150] Shenoy S Mohanakumar T Chatila T Tersak J Duffy B Wang R Defective apoptosis in lymphocytes and the role of IL-2 in autoimmune hematologic cytopenias. Clin Immunol (2001) 99(2):266–75. 10.1006/clim.2001.5017 11318598

[151] Olsson B Andersson PO Jacobsson S Carlsson L Wadenvik H. Disturbed apoptosis of T-cells in patients with active idiopathic thrombocytopenic purpura. Thromb Haemost (2005) 93(1):139–44. 10.1160/TH04-06-0385 15630504

[152] Olsson B Jernas M Wadenvik H Increased plasma levels of granzymes in adult patients with chronic immune thrombocytopenia. Thromb Haemost (2012) 107(6):1182–4. 10.1160/TH12-01-0012 22476618

[153] Zhang F Chu X Wang L Zhu Y Li L Ma D Cell-mediated lysis of autologous platelets in chronic idiopathic thrombocytopenic purpura. Eur J Haematol (2006) 76(5):427–31. 10.1111/j.1600-0609.2005.00622.x 16480433

[154] Zhao C Li X Zhang F Wang L Peng J Hou M Increased cytotoxic T-lymphocyte-mediated cytotoxicity predominant in patients with idiopathic thrombocytopenic purpura without platelet autoantibodies. Haematologica (2008) 93(9):1428–30. 10.3324/haematol.12889 18757854

[155] Qiu J Liu X Li X Zhang X Han P Zhou H CD8(+) T cells induce platelet clearance in the liver via platelet desialylation in immune thrombocytopenia. Sci Rep (2016) 6:27445. 10.1038/srep27445 27321376 PMC4913243

[156] Olsson B Ridell B Carlsson L Jacobsson S Wadenvik H. Recruitment of T cells into bone marrow of ITP patients possibly due to elevated expression of VLA-4 and CX3CR1. Blood (2008) 112(4):1078–84. 10.1182/blood-2008-02-139402 18519809

[157] Li S Wang L Zhao C Li L Peng J Hou M. CD8+ T cells suppress autologous megakaryocyte apoptosis in idiopathic thrombocytopenic purpura. Br J Haematol (2007) 139(4):605–11. 10.1111/j.1365-2141.2007.06737.x 17979946

[158] Audia S Samson M Mahevas M Ferrand C Trad M Ciudad M Preferential splenic CD8(+) T-cell activation in rituximab-nonresponder patients with immune thrombocytopenia. Blood (2013) 122(14):2477–86. 10.1182/blood-2013-03-491415 23963041

[159] Ma L Simpson E Li J Xuan M Xu M Baker L CD8+ T cells are predominantly protective and required for effective steroid therapy in murine models of immune thrombocytopenia. Blood (2015) 126(2):247–56. 10.1182/blood-2015-03-635417 26036802 PMC4505012

[160] Shlomchik MJ Craft JE Mamula MJ. From T to B and back again: positive feedback in systemic autoimmune disease. Nat Rev Immunol (2001) 1(2):147–53. 10.1038/35100573 11905822

[161] Guo L Kapur R Aslam R Speck ER Zufferey A Zhao Y CD20+ B-cell depletion therapy suppresses murine CD8+ T-cell-mediated immune thrombocytopenia. Blood (2016) 127(6):735–8. 10.1182/blood-2015-06-655126 26556550

[162] Li X Zhong H Bao W Boulad N Evangelista J Haider MA Defective regulatory B-cell compartment in patients with immune thrombocytopenia. Blood (2012) 120(16):3318–25. 10.1182/blood-2012-05-432575 22859611 PMC3476542

[163] Mauri C Menon M. Human regulatory B cells in health and disease: therapeutic potential. J Clin Invest (2017) 127(3):772–9. 10.1172/JCI85113 28248202 PMC5330739

[164] Veneri D Gottardi M Guizzardi E Zanuso C Krampera M Franchini M Idiopathic thrombocytopenic purpura, *Helicobacter pylori* infection, and HLA class II alleles. Blood (2002) 100(5):1925–6; author rely 6–7. 10.1182/blood-2002-05-1348 12211195

[165] Gouttefangeas C Diehl M Keilholz W Hornlein RF Stevanovic S Rammensee HG. Thrombocyte HLA molecules retain nonrenewable endogenous peptides of megakaryocyte lineage and do not stimulate direct allocytotoxicity in vitro. Blood (2000) 95(10):3168–75. 10807784

[166] Chapman LM Aggrey AA Field DJ Srivastava K Ture S Yui K Platelets present antigen in the context of MHC class I. J Immunol (2012) 189(2):916–23. 10.4049/jimmunol.1200580 22706078 PMC3392496

[167] Trugilho MRO Hottz ED Brunoro GVF Teixeira-Ferreira A Carvalho PC Salazar GA Platelet proteome reveals novel pathways of platelet activation and platelet-mediated immunoregulation in dengue. PLoS Pathog (2017) 13(5):e1006385. 10.1371/journal.ppat.1006385 28542641 PMC5453622

[168] Boshkov LK Kelton JG Halloran PF. HLA-DR expression by platelets in acute idiopathic thrombocytopenic purpura. Br J Haematol (1992) 81(4):552–7. 10.1111/j.1365-2141.1992.tb02991.x 1390243

[169] Fahim NM Monir E. Functional role of CD4+CD25+ regulatory T cells and transforming growth factor-beta1 in childhood immune thrombocytopenic purpura. Egypt J Immunol (2006) 13(1):173–87. 17974160

[170] Sakakura M Wada H Tawara I Nobori T Sugiyama T Sagawa N Reduced Cd4+Cd25+ T cells in patients with idiopathic thrombocytopenic purpura. Thromb Res (2007) 120(2):187–93. 10.1016/j.thromres.2006.09.008 17067661

[171] Liu B Zhao H Poon MC Han Z Gu D Xu M Abnormality of CD4(+)CD25(+) regulatory T cells in idiopathic thrombocytopenic purpura. Eur J Haematol (2007) 78(2):139–43. 10.1111/j.1600-0609.2006.00780.x 17328716

[172] Yu J Heck S Patel V Levan J Yu Y Bussel JB Defective circulating CD25 regulatory T cells in patients with chronic immune thrombocytopenic purpura. Blood (2008) 112(4):1325–8. 10.1182/blood-2008-01-135335 18420827 PMC2515134

[173] Zhang XL Peng J Sun JZ Liu JJ Guo CS Wang ZG De novo induction of platelet-specific CD4(+)CD25(+) regulatory T cells from CD4(+)CD25(-) cells in patients with idiopathic thrombocytopenic purpura. Blood (2009) 113(11):2568–77. 10.1182/blood-2008-03-148288 19056692

[174] Stasi R Cooper N Del Poeta G Stipa E Laura Evangelista M Abruzzese E Analysis of regulatory T-cell changes in patients with idiopathic thrombocytopenic purpura receiving B cell-depleting therapy with rituximab. Blood (2008) 112(4):1147–50. 10.1182/blood-2007-12-129262 18375792

[175] Bao W Bussel JB Heck S He W Karpoff M Boulad N Improved regulatory T-cell activity in patients with chronic immune thrombocytopenia treated with thrombopoietic agents. Blood (2010) 116(22):4639–45. 10.1182/blood-2010-04-281717 20688957 PMC2996119

[176] Audia S Samson M Guy J Janikashvili N Fraszczak J Trad M Immunologic effects of rituximab on the human spleen in immune thrombocytopenia. Blood (2011) 118(16):4394–400. 10.1182/blood-2011-03-344051 21876120 PMC3204911

[177] Li Z Mou W Lu G Cao J He X Pan X Low-dose rituximab combined with short-term glucocorticoids up-regulates Treg cell levels in patients with immune thrombocytopenia. Int J Hematol (2011) 93(1):91–8. 10.1007/s12185-010-0753-z 21188563

[178] Ling Y Cao X Yu Z Ruan C. Circulating dendritic cells subsets and CD4+Foxp3+ regulatory T cells in adult patients with chronic ITP before and after treatment with high-dose dexamethasome. Eur J Haematol (2007) 79(4):310–6. 10.1111/j.1600-0609.2007.00917.x 17692100

[179] Aslam R Hu Y Gebremeskel S Segel GB Speck ER Guo L Thymic retention of CD4+CD25+FoxP3+ T regulatory cells is associated with their peripheral deficiency and thrombocytopenia in a murine model of immune thrombocytopenia. Blood (2012) 120(10):2127–32. 10.1182/blood-2012-02-413526 22760780

[180] Catani L Sollazzo D Trabanelli S Curti A Evangelisti C Polverelli N Decreased expression of indoleamine 2,3-dioxygenase 1 in dendritic cells contributes to impaired regulatory T cell development in immune thrombocytopenia. Ann Hematol (2013) 92(1):67–78. 10.1007/s00277-012-1556-5 22936460

[181] Siragam V Crow AR Brinc D Song S Freedman J Lazarus AH. Intravenous immunoglobulin ameliorates ITP via activating Fc gamma receptors on dendritic cells. Nat Med (2006) 12(6):688–92. 10.1038/nm1416 16715090

[182] Kapur R Kim M Rebetz J Rondina MT Porcelijn L Semple JW Low levels of interleukin-10 in patients with transfusion-related acute lung injury. Ann Transl Med (2017) 5(16):339 10.21037/atm.2017.04.37 28861436 PMC5566739

[183] Kapur R Kim M Aslam R McVey MJ Tabuchi A Luo A T regulatory cells and dendritic cells protect against transfusion-related acute lung injury via IL-10. Blood (2017) 129(18):2557–69. 10.1182/blood-2016-12-758185 28202460 PMC5418638

[184] Houwerzijl EJ Blom NR van der Want JJ Esselink MT Koornstra JJ Smit JW Ultrastructural study shows morphologic features of apoptosis and para-apoptosis in megakaryocytes from patients with idiopathic thrombocytopenic purpura. Blood (2004) 103(2):500–6. 10.1182/blood-2003-01-0275 12969975

[185] Zhong H Bao W Li X Miller A Seery C Haq N CD16+ monocytes control T-cell subset development in immune thrombocytopenia. Blood (2012) 120(16):3326–35. 10.1182/blood-2012-06-434605 22915651 PMC3476543

[186] Liu XG Ma SH Sun JZ Ren J Shi Y Sun L High-dose dexamethasone shifts the balance of stimulatory and inhibitory Fcgamma receptors on monocytes in patients with primary immune thrombocytopenia. Blood (2011) 117(6):2061–9. 10.1182/blood-2010-07-295477 21131591

[187] Asahi A Nishimoto T Okazaki Y Suzuki H Masaoka T Kawakami Y *Helicobacter pylori* eradication shifts monocyte Fcgamma receptor balance toward inhibitory FcgammaRIIB in immune thrombocytopenic purpura patients. J Clin Invest (2008) 118(8):2939–49. 10.1172/JCI34496 18654664 PMC2483681

[188] Semple JW Bruce S Freedman J. Suppressed natural killer cell activity in patients with chronic autoimmune thrombocytopenic purpura. Am J Hematol (1991) 37(4):258–62. 10.1002/ajh.2830370409 1858783

[189] Garcia-Suarez J Prieto A Reyes E Manzano L Merino JL Alvarez-Mon M. Severe chronic autoimmune thrombocytopenic purpura is associated with an expansion of CD56+ CD3- natural killer cells subset. Blood (1993) 82(5):1538–45. 7689873

[190] Ebbo M Audonnet S Grados A Benarous L Mahevas M Godeau B NK cell compartment in the peripheral blood and spleen in adult patients with primary immune thrombocytopenia. Clin Immunol (2017) 177:18–28. 10.1016/j.clim.2015.11.005 26598010

[191] Koupenova M Clancy L Corkrey HA Freedman JE Circulating platelets as mediators of immunity, inflammation, and thrombosis. Circ Res (2018) 122(2):337–51. 10.1161/CIRCRESAHA.117.310795 29348254 PMC5777300

[192] Nishimoto T Numajiri M Nakazaki H Okazaki Y Kuwana M. Induction of immune tolerance to platelet antigen by short-term thrombopoietin treatment in a mouse model of immune thrombocytopenia. Int J Hematol (2014) 100(4):341–4. 10.1007/s12185-014-1661-4 25212676

[193] Ghadaki B Nazi I Kelton JG Arnold DM. Sustained remissions of immune thrombocytopenia associated with the use of thrombopoietin receptor agonists. Transfusion (2013) 53(11):2807–12. 10.1111/trf.12139 23451917 PMC3938454

[194] Thachil J Salter I George JN Complete remission of refractory immune thrombocytopenia (ITP) with a short course of Romiplostim. Eur J Haematol (2013) 91(4):376–7. 10.1111/ejh.12165 23822831

[195] Mahevas M Fain O Ebbo M Roudot-Thoraval F Limal N Khellaf M The temporary use of thrombopoietin-receptor agonists may induce a prolonged remission in adult chronic immune thrombocytopenia. Results of a French observational study. Br J Haematol (2014) 165(6):865–9. 10.1111/bjh.12888 24725224

[196] Grewal IS Flavell RA. CD40 and CD154 in cell-mediated immunity. Annu Rev Immunol (1998) 16:111–35. 10.1146/annurev.immunol.16.1.111 9597126

[197] Solanilla A Pasquet JM Viallard JF Contin C Grosset C Dechanet-Merville J Platelet-associated CD154 in immune thrombocytopenic purpura. Blood (2005) 105(1):215–8. 10.1182/blood-2003-07-2367 15191945

[198] Kuwana M Nomura S Fujimura K Nagasawa T Muto Y Kurata Y Effect of a single injection of humanized anti-CD154 monoclonal antibody on the platelet-specific autoimmune response in patients with immune thrombocytopenic purpura. Blood (2004) 103(4):1229–36. 10.1182/blood-2003-06-2167 14551140

[199] Kuwana M Kawakami Y Ikeda Y. Suppression of autoreactive T-cell response to glycoprotein IIb/IIIa by blockade of CD40/CD154 interaction: implications for treatment of immune thrombocytopenic purpura. Blood (2003) 101(2):621–3. 10.1182/blood-2002-07-2157 12393517

[200] Bruhns P Iannascoli B England P Mancardi DA Fernandez N Jorieux S Specificity and affinity of human Fcgamma receptors and their polymorphic variants for human IgG subclasses. Blood (2009) 113(16):3716–25. 10.1182/blood-2008-09-179754 19018092

[201] Kono H Kyogoku C Suzuki T Tsuchiya N Honda H Yamamoto K FcgammaRIIB Ile232Thr transmembrane polymorphism associated with human systemic lupus erythematosus decreases affinity to lipid rafts and attenuates inhibitory effects on B cell receptor signaling. Hum Mol Genet (2005) 14(19):2881–92. 10.1093/hmg/ddi320 16115811

[202] Li X Wu J Ptacek T Redden DT Brown EE Alarcon GS Allelic-dependent expression of an activating Fc receptor on B cells enhances humoral immune responses. Sci Transl Med (2013) 5(216):216ra175 10.1126/scitranslmed.3007097 PMC3982386 24353158

[203] Stegmann TC Veldhuisen B Nagelkerke SQ Winkelhorst D Schonewille H Verduin EP RhIg-prophylaxis is not influenced by FCGR2/3 polymorphisms involved in red blood cell clearance. Blood (2017) 129(8):1045–8. 10.1182/blood-2016-05-716365 28082442

[204] van Egmond M Vidarsson G Bakema JE. Cross-talk between pathogen recognizing toll-like receptors and immunoglobulin Fc receptors in immunity. Immunol Rev (2015) 268(1):311–27. 10.1111/imr.12333 26497530

[205] Schwab I Nimmerjahn F. Intravenous immunoglobulin therapy: how does IgG modulate the immune system? Nat Rev Immunol (2013) 13(3):176–89. 10.1038/nri3401 23411799

[206] Audia S Santegoets K Laarhoven AG Vidarsson G Facy O Ortega-Deballon P Fcgamma receptor expression on splenic macrophages in adult immune thrombocytopenia. Clin Exp Immunol (2017) 188(2):275–82. 10.1111/cei.12935 28142207 PMC5383444

[207] Podolanczuk A Lazarus AH Crow AR Grossbard E Bussel JB. Of mice and men: an open-label pilot study for treatment of immune thrombocytopenic purpura by an inhibitor of Syk. Blood (2009) 113(14):3154–60. 10.1182/blood-2008-07-166439 19096013

[208] Heitink-Polle KMJ Laarhoven AG Bruin MCA Veldhuisen B Nagelkerke SQ Kuijpers T Fc-gamma receptor polymorphisms are associated with susceptibility to and recovery from pediatric immune thrombocytopenia. Blood (2016) 128(22):867.

[209] Li J van der Wal DE Zhu G Xu M Yougbare I Ma L Desialylation is a mechanism of Fc-independent platelet clearance and a therapeutic target in immune thrombocytopenia. Nat Commun (2015) 6:7737. 10.1038/ncomms8737 26185093 PMC4518313

[210] Jansen AJ Josefsson EC Rumjantseva V Liu QP Falet H Bergmeier W Desialylation accelerates platelet clearance after refrigeration and initiates GPIbalpha metalloproteinase-mediated cleavage in mice. Blood (2012) 119(5):1263–73. 10.1182/blood-2011-05-355628 22101895 PMC3277358

[211] Li J Callum JL Lin Y Zhou Y Zhu G Ni H Severe platelet desialylation in a patient with glycoprotein Ib/IX antibody-mediated immune thrombocytopenia and fatal pulmonary hemorrhage. Haematologica (2014) 99(4):e61–3. 10.3324/haematol.2013.102897 24532041 PMC3971097

[212] Shao L Wu Y Zhou H Qin P Ni H Peng J Successful treatment with oseltamivir phosphate in a patient with chronic immune thrombocytopenia positive for anti-GPIb/IX autoantibody. Platelets (2015) 26(5):495–7. 10.3109/09537104.2014.948838 25166956

[213] Jansen AJ Peng J Zhao HG Hou M Ni H Sialidase inhibition to increase platelet counts: a new treatment option for thrombocytopenia. Am J Hematol (2015) 90(5):E94–5. 10.1002/ajh.23953 25615710

[214] Alioglu B Tasar A Ozen C Selver B Dallar Y An experience of oseltamivir phosphate (tamiflu) in a pediatric patient with chronic idiopathic thrombocytopenic purpura: a case report. Pathophysiol Haemost Thromb (2010) 37(2–4):55–8. 10.1159/000321379 21063076

[215] Tao L Zeng Q Li J Xu M Wang J Pan Y Platelet desialylation correlates with efficacy of first-line therapies for immune thrombocytopenia. J Hematol Oncol (2017) 10(1):46. 10.1186/s13045-017-0413-3 28179000 PMC5304552

[216] Kapur R Heitink-Polle KM Porcelijn L Bentlage AE Bruin MC Visser R C-reactive protein enhances IgG-mediated phagocyte responses and thrombocytopenia. Blood (2015) 125(11):1793–802. 10.1182/blood-2014-05-579110 25548320

[217] Zhang B Lo C Shen L Sood R Jones C Cusmano-Ozog K The role of vanin-1 and oxidative stress-related pathways in distinguishing acute and chronic pediatric ITP. Blood (2011) 117(17):4569–79. 10.1182/blood-2010-09-304931 21325602

[218] Jin CQ Dong HX Cheng PP Zhou JW Zheng BY Liu F. Antioxidant status and oxidative stress in patients with chronic ITP. Scand J Immunol (2013) 77(6):482–7. 10.1111/sji.12048 23551069

[219] Elsalakawy WA Ali MA Hegazy MG Farweez BA. Value of vanin-1 assessment in adult patients with primary immune thrombocytopenia. Platelets (2014) 25(2):86–92. 10.3109/09537104.2013.782484 23534352

[220] Premsler T Lewandrowski U Sickmann A Zahedi RP. Phosphoproteome analysis of the platelet plasma membrane. Methods Mol Biol (2011) 728:279–90. 10.1007/978-1-61779-068-3_19 21468956

[221] Lee-Sundlov MM Ashline DJ Hanneman AJ Grozovsky R Reinhold VN Hoffmeister KM Circulating blood and platelets supply glycosyltransferases that enable extrinsic extracellular glycosylation. Glycobiology (2017) 27(2):188–98. 10.1093/glycob/cww108 27798070 PMC5224594

[222] Fong KP Barry C Tran AN Traxler EA Wannemacher KM Tang HY Deciphering the human platelet sheddome. Blood (2011) 117(1):e15–26. 10.1182/blood-2010-05-283838 20962327 PMC3037762

[223] Andrews RK Gardiner EE. Basic mechanisms of platelet receptor shedding. Platelets (2017) 28(4):319–24. 10.1080/09537104.2016.1235690 27778531

[224] Au AE Josefsson EC. Regulation of platelet membrane protein shedding in health and disease. Platelets (2017) 28(4):342–53. 10.1080/09537104.2016.1203401 27494300

[225] Doyle HA Mamula MJ. Post-translational protein modifications in antigen recognition and autoimmunity. Trends Immunol (2001) 22(8):443–9. 10.1016/S1471-4906(01)01976-7 11473834

[226] Zavala-Cerna MG Martinez-Garcia EA Torres-Bugarin O Rubio-Jurado B Riebeling C Nava A. The clinical significance of posttranslational modification of autoantigens. Clin Rev Allergy Immunol (2014) 47(1):73–90. 10.1007/s12016-014-8424-0 24840362

[227] Klareskog L Ronnelid J Lundberg K Padyukov L Alfredsson L. Immunity to citrullinated proteins in rheumatoid arthritis. Annu Rev Immunol (2008) 26:651–75. 10.1146/annurev.immunol.26.021607.090244 18173373

